# Putting theory to the test: An integrated computational/experimental chemostat model of the tragedy of the commons

**DOI:** 10.1371/journal.pone.0300887

**Published:** 2024-04-10

**Authors:** Bryan K. Lynn, Patrick De Leenheer, Martin Schuster

**Affiliations:** 1 Department of Integrative Biology, Oregon State University, Corvallis, Oregon, United States of America; 2 Department of Mathematics, Oregon State University, Corvallis, Oregon, United States of America; 3 Department of Microbiology, Oregon State University, Corvallis, Oregon, United States of America; Federal University Dutse, NIGERIA

## Abstract

Cooperation via shared public goods is ubiquitous in nature, however, noncontributing social cheaters can exploit the public goods provided by cooperating individuals to gain a fitness advantage. Theory predicts that this dynamic can cause a Tragedy of the Commons, and in particular, a ‘Collapsing’ Tragedy defined as the extinction of the entire population if the public good is essential. However, there is little empirical evidence of the Collapsing Tragedy in evolutionary biology. Here, we experimentally demonstrate this outcome in a microbial model system, the public good-producing bacterium *Pseudomonas aeruginosa* grown in a continuous-culture chemostat. In a growth medium that requires extracellular protein digestion, we find that *P*. *aeruginosa* populations maintain a high density when entirely composed of cooperating, protease-producing cells but completely collapse when non-producing cheater cells are introduced. We formulate a mechanistic mathematical model that recapitulates experimental observations and suggests key parameters, such as the dilution rate and the cost of public good production, that define the stability of cooperative behavior. We combine model prediction with experimental validation to explain striking differences in the long-term cheater trajectories of replicate cocultures through mutational events that increase cheater fitness. Taken together, our integrated empirical and theoretical approach validates and parametrizes the Collapsing Tragedy in a microbial population, and provides a quantitative, mechanistic framework for generating testable predictions of social behavior.

## Introduction

Cooperation is a behavior that contributes to the benefit of another individual or group but has a fitness cost for the individual. Cooperation is necessary for the functioning of many, if not all, biological systems across a range of organismal complexity—from the simpler bacterial systems to the more complex human societies [1–4]. There are many different ways organisms can cooperate, such as the responsible use of shared resources, contributing to a common goal, and refraining from competition with one another [5]. Resources shared in cooperative societies are often referred to as “public goods,” and one challenge these societies face when managing public goods is the emergence of social cheaters [2, 4, 6]. Cheaters benefit from the cooperation of others without contributing to the associated cost, resulting in a fitness advantage and proliferation throughout the population. This tension between maximizing the fitness of the individual and the fitness of the group may result in a phenomenon known as the Tragedy of the Commons, and is named such because once enough cheaters have invaded the population, the fitness of the group significantly reduces [7]. If the public good is essential to cooperation, then cheaters may invade to the point of catalyzing a complete population collapse, a scenario referred to as a Collapsing Tragedy [5].

Validating existing mathematical theory through experimentation on cooperation and the Tragedy of the Commons can be challenging, because although several different mathematical models have previously been used to describe cooperative behaviors, many are phenomenological in nature—meaning they can describe what happens but not why it happens and lack integration of biological processes [4, 8–11]. This has resulted in calls to shift towards a mechanistic modeling approach with a greater integration of empirical data in the study of cooperation [4, 9, 12–14]. Mathematical modeling of chemostats, continuous microbial culturing systems, are mechanistic by design, allowing for such an integration of these biological processes and, thus, a better understanding of their role in the system dynamics. Chemostat theory of cooperation predicts: (i) there will be a stable population when there are no cheaters present, (ii) there will be a Tragedy of the Commons when cheaters are introduced, and (iii) the frequency of cheaters in the population will increase throughout cultivation [15]. Since the Tragedy of the Commons of cooperative microbial populations in a chemostat has been theoretically predicted, this makes the chemostat an ideal candidate for examining the extent to which the mathematical theory holds up in empirical conditions.

In addition to the theoretical predictions, experiments with social microbes have inferred the Tragedy of the Commons from relative fitness measurements [16–18], but very few experiments have directly demonstrated the population collapse as the ultimate outcome. The absence of direct demonstration of a Collapsing Tragedy is often simply a byproduct of methodological limitations. When using standard microbial growth conditions, such as on an agar plate or in classic batch culture (as in [16] and [18] respectively), the cell density continuously increases to a point of saturation when all of the nutrients have been consumed. In these settings, cheaters can invade and potentially cause growth arrest of the population, but they will not eliminate the population altogether. As such, the use of relative fitness measurements to infer the Tragedy of the Commons has become widespread and applied even when the methods would allow it to be observed (as in [17]). Rare examples in which a Collapsing Tragedy was observed include fruiting body development in *Myxococcus xanthus* where signaling-deficient cheaters abrogate spore production [19], and biofilm formation in *Pseudomonas fluorescens*, where social cheaters not contributing to the structure of the biofilm caused the mat of aggregating cells to prematurely fall apart [20]. Verifying that a Collapsing Tragedy of the Commons occurs in microbial populations is both beneficial and necessary because: (i) it provides the opportunity to validate that the existing mathematical theory sufficiently encapsulates the dynamics of the system, (ii) it allows us to identify the predictive limitations of such models, and (iii) it is not uncommon for cooperative populations to evade collapse as a consequence of mechanisms that either increase the cooperator’s fitness or decrease the cheater’s [19, 21, 22]. Examples of such mechanisms in microbial populations include non-social adaptations [23, 24], punishment of cheaters through the production of toxic substances [25–27], reciprocity [28], privatization of public goods [29–31], division of labor to reduce individual fitness costs of beneficial behaviors [32–34], horizontal gene transfer [35, 36], or population structure [17, 18, 37, 38]. By identifying empirical conditions under which the Tragedy of the Commons occurs, we can better understand the conditions necessary to avoid it.

*Pseudomonas aeruginosa* is an example of a bacterium that exhibits a range of cooperative behaviors, generally in the form of secreted public goods that are shared within the population [3, 4, 39]. Examples of extracellular public goods include proteases necessary for metabolizing food sources, biosurfactants for swarming, and siderophores for iron scavenging [3]. More specifically, an example of an extracellular protease produced by *P*. *aeruginosa* is LasB elastase, which is necessary to digest protein substrates [23, 39–42]. LasB and many other genes that encode cooperative functions are controlled by a process called quorum sensing (QS) [42, 43]. QS regulates gene expression in response to population density via diffusible chemical signals [44, 45]. In *P*. *aeruginosa*, the central QS regulator LasR binds a specific acyl-homoserine lactone signal and activates transcription of target genes. A mutation in the *lasR* gene results in “signal-blind” cells that do not respond to the QS signal, eliminating the expression of proteases and other products. These *lasR* mutants are obligatory cheaters: they cannot grow on their own without the proteases, but they have a growth advantage when grown with the cooperating parent strain. This growth advantage results from the loss of the metabolic cost associated with producing the proteases while still benefiting from the presence of proteases produced by other cells [18, 41, 46].

In this study, we combined empirical and theoretical approaches to test the prediction that a Collapsing Tragedy of *P*. *aeruginosa* populations occurs in the chemostat. We conducted a series of growth experiments in the chemostat under conditions that require QS-dependent proteolysis (the breakdown of proteins by enzymes produced via QS) involving two strains: (i) the cooperating wild type (WT) strain which has its QS circuitry intact and (ii) the cheating *lasR* mutant which cannot respond to QS signals and thus does not produce any costly proteases. In addition to comparing our data to the general outcomes predicted by chemostat theory, we further aim to assess the predictive capabilities of such models. To accomplish this, we use our experimental conditions to inform the construction and analysis of a mechanistic mathematical model that specifically describes our system. We then fit the model to our data and applied it to make experimentally testable predictions. By employing both experimentation and mechanistic mathematical modeling, we demonstrate that the Tragedy of the Commons is indeed the outcome of social cheating in a well-mixed population; however, the theory only holds for population-level predictions. The deterministic model was unable to capture subpopulation dynamics, but reconciled those differences by incorporating mutational events into the model that could be experimentally validated.

## Methods

### Bacterial strains

In this study, we used the following strains: *P*. *aeruginosa* PAO1 WT strain and its isogenic *lasR* deletion mutant [41, 46]. The *lasR* strain carries a stable trimethoprim antibiotic-resistance gene cassette at a neutral chromosomal site (mini-Tn7-Tp) that allows distinction from the WT in coculture and does not affect growth [46–48].

### Cultures and growth conditions

*P*. *aeruginosa* cultures were routinely grown at 37°C in lysogeny broth (LB) liquid or plate culture buffered with 50 mM 3-(*N*-morpholino)-propanesulfonic acid (MOPS), pH 7.0. Liquid batch cultures were shaken at 250 rotations per minute (RPM). Growth was measured as either colony forming units (CFU) or as optical density at 600 nm (OD_600_).

Pre-cultures were grown in LB-MOPS liquid culture for approximately 18 hours. Experimental cultures were grown in M9-gelatin medium, either in a batch or chemostat environment. The medium contained 1 x M9 salts, 1% (w/v) gelatin, 1 mM MgSO_4_ 1M CaCl_2_, and 1000x non-chelated trace elements. Type B (powder, Sigma Aldrich, G9391) gelatin was used for the initial batch cultures, in which the WT and *lasR* mutant strains were grown independently and then again as a coculture. For the remaining cultures, the gelatin used was of type A (10% solution, Alfa Aesar, J62699). The switch from type B gelatin used initially to the type A gelatin was due to the volume of medium required for those experiments. Making gelatin medium with type B powder required sterile filtration, and the filters were not practical for large scales of medium. Cultures were either mono or cocultures (WT or *lasR* mutant, or a mixture of both), inoculated with the respective pre-culture to a starting total OD_600_ of 0.05.

#### Batch cultures

The batch mono and cocultures were grown in flasks containing 20 mL of M9-gelatin medium for 36 to 48 hours, as indicated. The cocultures had an initial *lasR* mutant frequency of 1%. The batch culture used to determine the correlation between CFU/mL and OD_600_ values was grown in flasks containing 30 mL of M9-gelatin medium for 42 hours. For each batch culture, three biological replicates were performed.

To determine the frequency of *lasR* mutants in cocultures, 10 μL of a diluted culture sample was spotted six times onto a standard LB plate and an LB plate with 200 μg/mL trimethoprim. The standard LB plate was placed in a 30°C incubator while the trimethoprim plate was placed in a 37°C incubator. The different incubation temperatures were used to compensate for the decreased bacterial growth rate on the antibiotic plates. Colonies were counted approximately 24 hours later. The frequency of *lasR* mutant cells was calculated by determining the average CFU per ml on each type of medium.

#### Chemostat cultures

The chemostat cultures were grown in 100 mL of M9-gelatin medium, using a chemostat bioreactor system previously designed in our laboratory [49, 50]. It contains a substrate inflow controlled by a peristaltic pump, a culture outflow, and an air supply from an aquarium pump. It is operated in a 37°C room and utilizes a stir bar to achieve a well-mixed and well-aerated bacterial culture. The chemostat was inoculated with the pre-culture to a starting OD_600_ of 0.05. Two biological replicates were performed for the WT-only chemostat experiment, and four biological replicates were performed for the coculture chemostat experiment. We will refer to the coculture replicates as Replicates 1 to 4 throughout.

The chemostat cultures were first grown in the bioreactor close to saturation, with the medium supply pump turned off (31–32 hours for the WT monoculture and 49–56 hours for the WT:*lasR* coculture). Then the peristaltic pump was turned on to 2.75 RPM, corresponding to a flow rate of 12 ml/h. The chemostat was run for 183–192 hours, or approximately 8 days. For both the monocultures and cocultures, OD_600_ was taken periodically. For the cocultures, plating on differential medium was used to determine the frequency of *lasR* mutant and protease-deficient phenotypes [41, 51].

#### Determining antibiotic-resistant and protease-deficient phenotypes

To determine the frequency of *lasR* and proteolysis-deficient mutants, chemostat samples were diluted and 100 μL of the diluted chemostat sample was spread onto a standard LB plate. The plate was placed in a 37°C incubator for approximately 18 hours to obtain small colonies. During chemostat cultivation, colony size heterogeneity occurred. From these initial sample plates we categorized the colonies into one of two groups based on size: small or regular. This categorization was purely methodological, allowing us to differentiate the incubation times between the two groups so the phenotypic expression could be properly analyzed. We counted the number of small and regular colonies on the plate determining their frequency within the population for cheater frequency analysis.

We then patched up to 100 randomly selected colonies of each size variant onto both skim milk and antibiotic plates. These skim milk plates contain 4% (w/v) skim milk powder (Difco Skim Milk, BD, 232100), 1.5% (w/v) agar, and 0.5% (w/v) LB. The antibiotic plates are a standard LB plate with 200 μg/mL trimethoprim added. One WT and one *lasR* mutant colony selected from an LB plate was added as a control. All plates were placed in a 37°C incubator for the following incubation times: 8 hours for the skim milk plates with regular colonies, 18 hours for the antibiotic plates with regular colonies and the skim milk plates with small colonies, and 28 hours for the antibiotic plates with the small colonies. The different incubation times were used so that the CFUs grew to a similar size prior to counting.

Antibiotic plates were evaluated upon removal from the incubator; skim milk plates were evaluated after an additional 20 hours of incubation in a fridge. The additional incubation in fridge allowed the enzyme to continue to degrade the milk while limiting additional bacterial growth. Protease-deficiency of individual isolates was determined based on the inability to form a large halo (a dark translucent circle indicating milk had been degraded) around the colony, as compared to defined WT and defined *lasR* mutant controls. Antibiotic resistance was determined by the presence of microbial growth on the antibiotic plate, indicative of the trimethoprim-resistant *lasR* mutant. With these data, the frequency of antibiotic-resistant (*lasR*-deficient) and protease-deficient isolates was calculated for each subpopulation (small vs. regular colony variants). We then combined the subpopulation phenotype frequencies with the frequency of each subpopulation in the total population to calculate the estimated phenotypic frequencies for the population as a whole.

#### Evolved mutant analysis

We investigated the relative fitness of *P*. *aeruginosa* mutants that evolved during chemostat cultivation. We focused on evolved cheater mutants we predicted to have occurred in coculture Replicates 1, 3, and 4, and compared their relative fitness to the original *lasR* mutant. Replicate 2 was not included in this analysis because it behaved differently from the other replicates making investigation into protease deficient mutants not necessary. An in-depth rationale for omitting Replicate 2 can be found below. Evolved mutants were isolated (i.e. separated as individual clones from evolved chemostat populations) by evaluating their skim-milk proteolysis and antibiotic-resistance phenotypes using the methods described in the previous section. Isolates were chosen according to the phenotype that matched the type of mutation predicted; for coculture Replicates 1 and 3 we selected mutants that were antibiotic-resistant (grew on the trimethoprim plate) and did not produce protease (did not degrade the skim milk). Of those mutants, we selected three isolates which had formed the largest colonies on the skim milk plate, indicative of a faster growth rate, to decrease the chances of selecting a defined *lasR* mutant with no additional mutations. For coculture Replicate 4 we selected three isolates that were not antibiotic-resistant (did not grow on the trimethoprim plate) and did not produce protease. For each coculture replicate experiment, three isolates of the matching phenotype were pre-cultured in LB-MOPS liquid medium for approximately 18 hours, along with the WT and the defined *lasR* mutant. Cocultures of each mutant (3 chemostat isolates and the defined *lasR* mutant) with the WT were grown as three replicates each in 4 mL of 1% M9 gelatin medium. The cocultures each had an initial mutant starting frequency of 1%. Replicates were grown for 36 hours in a 37°C incubator. Samples from the cocultures were taken at the time of inoculation and after 36 hours of growth. The frequency of protease-deficiency of these coculture samples was determined by skim milk proteolysis, as described in the previous paragraph.

#### Calculating relative fitness and statistical analysis

To calculate relative fitness of the *lasR* mutant in batch coculture, we first calculated the absolute fitness values of the *lasR* mutant and the WT as the average growth rate of each strain. Absolute fitness was based on CFU data collected at 0 and 36 hours of cultivation to determine cell densities (CFU/ml). We determined the *lasR* mutant cell density from growth on an antibiotic plate, and, to determine the WT cell density, we subtracted the cell density of the *lasR* mutants from the cell density of the entire coculture. Absolute fitness was calculated as the natural log of the ratio of the final and initial cell densities: $\ln\left(\frac{final\;cell\;density}{initial\;cell\;density}\right)$. To calculate the relative fitness from the absolute fitness calculations, we took the ratio of the *lasR* and WT absolute fitness values:

$$\mathrm{Relative}\;\mathrm{fitness}=\frac{\mathrm{Absolute}\;\mathrm{fitness}\;lasR}{\mathrm{Absolute}\;\mathrm{fitness}\;\mathrm{WT}}.$$

To determine the relative fitness for the evolved mutant cocultures, we followed the same approach as above but used milk plates to identify the ratio of cooperators and cheaters. This also eliminated the need to subtract the mutant population from the total population, since each subpopulation is counted independently.

Statistical analysis was done using GraphPad Prism 9.4.1.

#### Mathematical model formulation

**Fig 1:**
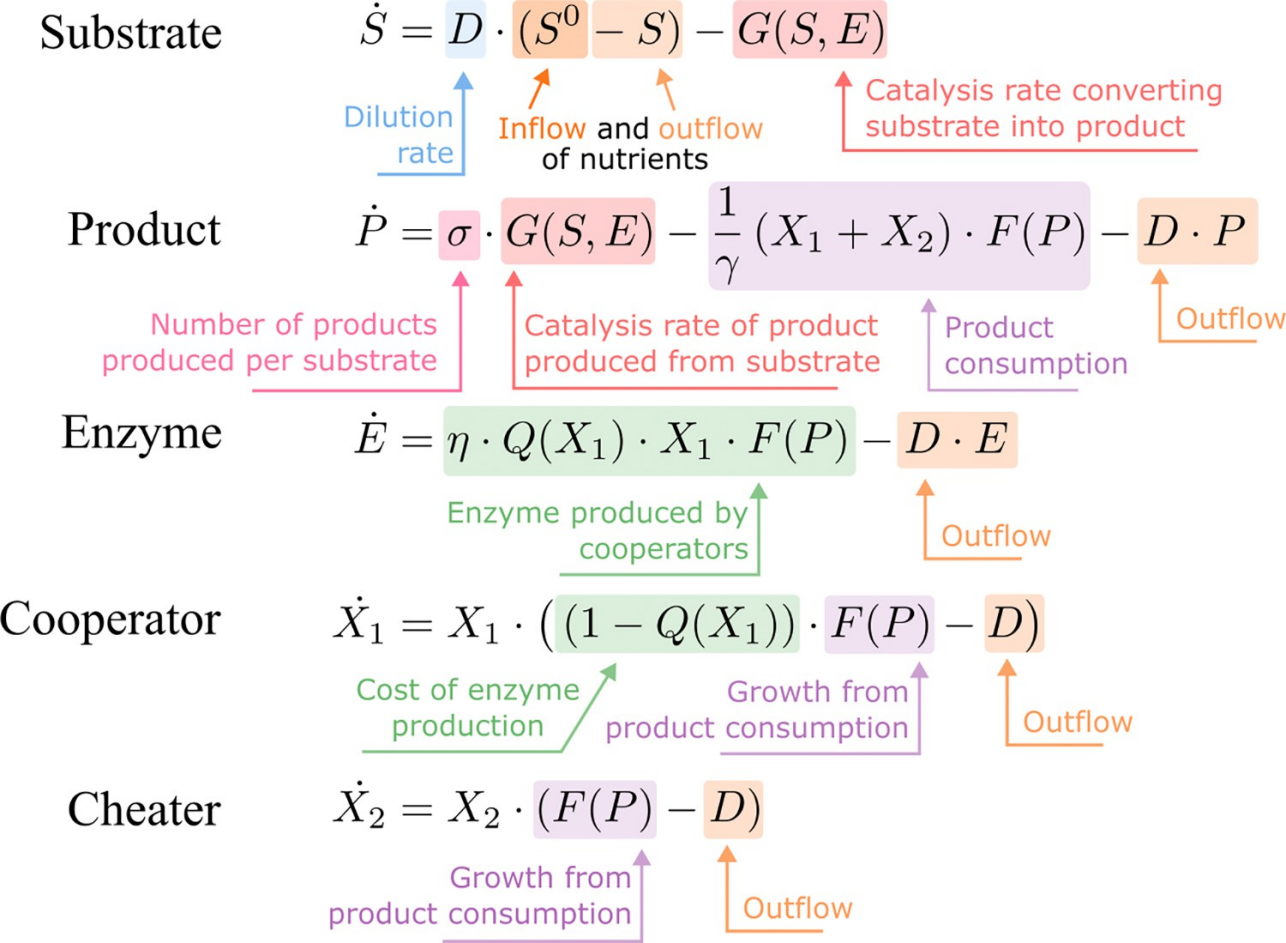
Annotated chemostat model. Chemostat model describing the change in concentrations of gelatin substrate (*S*), degradation product of the substrate (*P*), protease enzyme produced by the cooperator (*E*), the enzyme-producing bacterial cooperator (*X*_*1*_), and the bacterial cheater which does not produce any proteases (*X*_*2*_). A detailed description of the parameter definitions and units can be found in **Table 1**.

We used a system of ODEs to describe the changes of concentrations occurring within the chemostat while also considering the biological and physical processes that are taking place [15, 34, 52, 53]. Here *S* (mmol/l) is the proteinaceous substrate concentration, in this case gelatin, in the chemostat vessel and *S*^*0*^ (mmol/l) is the concentration of gelatin entering the system, *E* (mmol/l) is the protease enzyme, *P* (mmol/l) is the product of the enzymatic degradation of gelatin, *X*_*1*_ (g dry weight/l) is the cooperative WT strain, and *X*_*2*_ (g dry weight/l) is the protease-deficient mutant cheater strain. For simplicity, we considered a single extracellular protease that degrades gelatin into utilizable individual amino acids, recognizing that in reality there are several proteases that contribute to this process [54]. The model is as described in **Fig 1**, and a detailed parameter explanation follows.

*D* (1/h) is the dilution rate, meaning it is both the rate at which gelatin medium enters the chemostat and the rate at which the well-mixed culture is removed from the system. Experimentally, it is calculated by dividing the flow rate by the volume of the culture within the chemostat. Thus, the positive expression *DS*^*0*^ in the substrate equation adds gelatin substrate to the chemostat, and each expression containing a–*D* removes substrate, enzyme, product, and bacteria from the chemostat. In the enzymatic degradation of protein substrate, σ represents the number of cleavage or breakdown products generated per substrate molecule, η (mmol/g dry weight) is the amount of enzyme produced per bacterial biomass, and γ (g dry weight/mmol) is a yield constant that relates the product to bacterial biomass.

We also consider the Hill function *Q*(*X*_*1*_) as the fraction of metabolic energy spent on producing protease enzyme. It is dependent on the cell density of the WT strain being sufficiently high to induce QS gene expression. We chose a Hill function due to its switch-like properties and previous use to model cell to cell communication [55–57]. It reflects the cooperativity of QS with positive feedback on signal production and with receptor dimerization [55–57].

**Fig 2:**
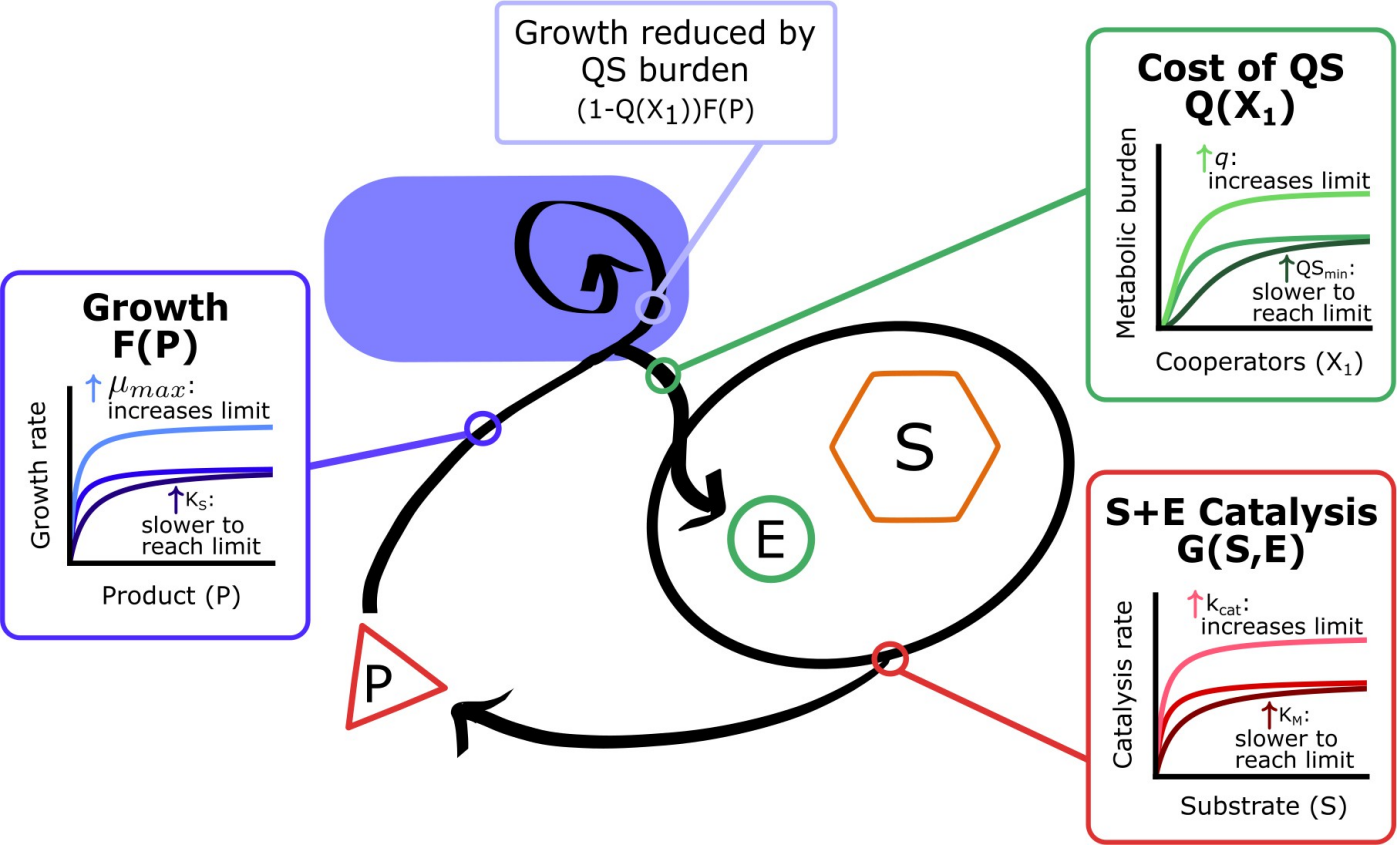
Overview of system and functional effects. Diagram of the metabolic system for an enzyme-producing cooperator. The cell produces enzyme (*E*) at a metabolic cost (*Q*(*X*_*1*_)), reducing its growth. The enzyme catalyzes the breakdown of substrate (*S*) into digestible product (*P*) at rate *G*(*S*,*E*). The cooperator grows at rate *F*(*P*), which is reduced by a fraction depending on the cost of enzyme production. The effect of increasing the parameters within each function are illustrated.

The growth of the cooperative strain is reduced by the fraction (1–*Q*(*X*_*1*_)) of the total possible growth experienced by the non-protease producing strain. The Hill function is given by

$$Q(X_{1})=\frac{q{∙(X_{1})}^{n}}{{\left(X_{1}\right)}^{n}+{\left(QS_{min}\right)}^{n}}$$

where *q* is the estimated burden from enzyme production when the entire population is QS-activated, *QS*_*min*_ is the cell density at which QS is turned on, and *n*, the Hill coefficient, determines the speed of transition from little metabolic burden to a metabolic burden of *q*. Thus, when cooperators are absent, *Q*(*X*_1_) will be equal to 0 (when *X*_1_ = 0, *Q*(*X*_1_) = 0), but, as the WT density increases so too will *Q*(*X*_1_), eventually approaching *q* (when *X*_1_ is very large, then *Q*(*X*_1_) asymptotically approaches *q*) (Fig 2).

We further considered two additional rate functions: *G*(*S*,*E*) is the cleavage rate of proteins by the secreted proteases and *F*(*P*) is the per capita growth rate of the organism. *G*(*S*,*E*) and *F*(*P*) are saturating Michaelis-Menten and Monod functions, respectively. It is worth noting that these are fundamentally the same type of function but are separately named due to the type of biological process being modeled. The Michaelis-Menten equation for enzyme kinetics has been studied for over a century and, in the past few decades, supported experimentally [58, 59]. In particular, there is experimental evidence validating the Michaelis-Menten equation with *P*. *aeruginosa* produced protease enzymes [42]. The Monod growth rate function was deduced empirically in conjunction with bacterial population growth [60] and has been shown to correctly model the growth of *P*. *aeruginosa* populations [42].

The enzymatic activity is defined as

$$G(S,E)=\frac{k_{cat}∙E∙S}{K_{M}+S}.$$

Here, *k*_*cat*_ (1/h), typically referred to as the turnover number, is a rate defined as the maximum number of substrate molecules converted to product by an enzyme molecule per unit time, and *K*_*M*_ (mmol/l), the Michaelis constant, is the gelatin concentration at half-maximal velocity. The rate of bacterial growth is given by

$$F(P)=\frac{\mu_{max}∙P}{K_{S}+P}.$$

For this growth rate equation, μ_max_ (1/h) is the maximum growth rate of *P*. *aeruginosa* and *K*_*S*_ (mmol/l) is the product concentration at the half-maximal growth rate.

Each parameter influences Michaelis-Menten and Monod functions such as *G*(*S*,*E*) and *F*(*P*) differently, with the parameters in the numerator (*k*_cat_ and μ_max_) impacting the overall magnitude of the function limit and the parameters in the denominator (*K*_*M*_ and *K*_*S*_) influencing the speed at which the function reaches its saturation limit.

#### Model parameterization and data fitting

Parameters and initial quantities were either experimentally determined by us or were based on values obtained from other sources (see S1 File for parameter derivations). To be suitable for our model, experimentally determined cell densities were converted from OD_600_ to grams of dry weight per liter (g dry weight/l) using a previously established conversion factor [50]. The model is well-posed in the sense that for all positive and real initial conditions, solutions remain positive, real, and bounded (see S2 File for proof). Not only is this biologically relevant, since the chemostat cannot hold an infinite amount of bacterial mass, it also indicates that the model will be less susceptible to instability and error magnification when solved numerically as compared to models which do not share those characteristics. The model was simulated in Python 3.9 using the ODEINT function of the Scipy package. To reflect our experimental design, the model was first simulated in batch mode (*D* = 0), and then in chemostat mode (*D* > 0).

When comparing the simulated populations to experimental data, we calculated the root mean square error (RMSE) of each replicate, then took the average of the RMSE values across all replicates of a given data type. To determine which values within our given estimated parameter value ranges is the best fit, we calculated the average RMSE of the simulated model against three experimental data sets: the total cell density of the WT-only culture, the total cell density of the coculture, and the cheater frequency determined by protease-deficiency of the coculture—omitting the data from Replicate 2 whose data diverged from the other three replicates. Because the cell density and cheater frequency data are on different scales, we calculated a normalized combined RMSE for each successful simulation by utilizing max-min normalization for each data type, then taking a weighted sum. We weighted the normalized RMSE values because parameter values which created higher cheater frequencies also created a higher final coculture batch phase cell density resulting in a best fit that greatly overshot the coculture batch phase growth. The normalized RMSE values were weighted for the WT-only, coculture, and cheater frequency data as 0.4, 0.6, and 0.2, respectively.

All parameters were randomly determined for each simulation, with two exceptions: the dilution rate (*D*) and the Hill coefficient (*n*). These parameters remain as one value throughout because the dilution rate is determined by experimental design and is conclusively one fixed rate and the effect of increasing the Hill coefficient beyond 2 is negligible to the dynamics of the system. Randomly determined initial condition and parameter values were selected from different distributions depending on whether the estimated value was a range of values or a single value. For the former we used a uniform distribution across their estimated range, and for the latter we used a normal distribution with the estimated single value as the mean and a standard deviation of 10% of the mean value. A simulation was deemed to successfully capture the general dynamics of our data if: (i) the solution had a positive WT cell density at the final time point (greater than 0.01 g dry weight/l or 0.0048 OD), eliminating parameter combinations that cause the cooperator-only population to wash out, (ii) a reasonable maximum coculture batch growth (less than 0.58 OD_600_) was achieved, removing parameter combinations that simulate excessive growth in coculture batch mode, and (iii) there was a cheater frequency of at least 30%, ignoring simulations where the cheater frequency fell to or near 0% (which occurs when the growth of the WT is sufficiently slow that they never achieve the minimum density required for QS, the dilution causes a washout of the system, and/or the total density of the population gets within machine error of 0, thus rounding to 0 and resulting in a cheater frequency calculation error).

To reduce the scope of the parameter and initial conditions to explore when determining the best fit, we first ran 100,000 simulations to establish if the entirety of each estimated parameter range can produce a simulation that successfully captures the general dynamics of the chemostat data (S1 File). Most parameters were capable of successful simulations across the entirety of their ranges; however, we were able to establish a new lower bound for η and *k*_*cat*_. We then ran 1,000,000 simulations with two changes: (i) we set the ranges of η and *k*_*cat*_ to 4x10^-4^ and 150 respectively, and (ii) we used the single estimated values instead of randomly selected values from a normal distribution since pulling from the normal distribution did not seem to impact success in the previous round of simulations. This produced 47631 simulations that captured the broad patters of our data.

Because the RMSE weights were chosen arbitrarily, we needed to investigate the simulations for goodness of fit rather than just taking the lowest weighted value. Of the 47631 simulations, we kept the 10% with the lowest combined weighted and normalized RMSE values. We further narrowed down that list by omitting simulations in which values at the end of the batch phase (time = 0) were outside of the range for the maximum and minimum coculture cell density and under 30% cheater frequency. This left us with 15 results to compare visually. After selecting the one with the best visual fit, we slightly adjusted individual or pairs of parameters to decrease the RMSE values until changes no longer resulted in a reduced RMSE (Fig 6). See S1 File for a comparison between the simulation with the lowest weighted and normalized combined RMSE.

#### Modified models for evolved mutants

In addition to experimentally exploring the role of the mutations that arose during cultivation, we also investigated their impact on the system computationally. The following systems of equations describe these evolved faster-growing mutants as

(i) a cheater derived from the defined *lasR* ancestor

$$\begin{array}{l} \dot{S}=D∙(S^{0}-S)-G(S,E)\\ \dot{P}=\sigma ∙G(S,E)-\frac{1}{\gamma }(\left(X_{1}+X_{2})∙F(P)+X_{3}{∙F}_{2}(P\right))-D∙P\\ \dot{E}=\eta ∙Q(X_{1})∙X_{1}∙F(P)-D∙E\\ {\dot{X}}_{1}=X_{1}∙((1-Q(X_{1}))∙F(P)-D)\\ {\dot{X}}_{2}=X_{2}∙(F(P)-D-\alpha )\\ {\dot{X}}_{3}=X_{3}∙(F_{2}(P)-D)+\alpha ∙X_{2}, \end{array}$$

(ii) a cheater derived from the WT ancestor

$$\begin{array}{l} \dot{S}=D∙(S^{0}-S)-G(S,E)\\ \dot{P}=\sigma ∙G(S,E)-\frac{1}{\gamma }∙(\left(X_{1}+X_{2})∙F(P)+X_{3}{∙F}_{2}(P\right))-D∙P\\ \dot{E}=\eta ∙Q(X_{1})∙X_{1}∙F(P)-D∙E\\ {\dot{X}}_{1}=X_{1}∙((1-Q(X_{1}))∙F(P)-D-\alpha )\\ {\dot{X}}_{2}=X_{2}∙(F(P)-D)\\ {\dot{X}}_{3}=X_{3}∙(F_{2}(P)-D)+\alpha ∙X_{1}, \end{array}$$

and (iii) a cooperator derived from the WT ancestor

$$\begin{array}{l} \dot{S}=D∙(S^{0}-S)-G(S,E)\\ \dot{P}=\sigma ∙G(S,E)-\frac{1}{\gamma }(\left(X_{1}+X_{2})∙F(P)+X_{3}∙F_{2}(P\right))-D∙P\\ \dot{E}=\eta ∙Q(X_{1}+X_{3})∙(X_{1}∙F(P)+X_{3}{∙F}_{2}(P))-D∙E\\ {\dot{X}}_{1}=X_{1}∙((1-Q(X_{1}+X_{3}))∙F(P)-D-\alpha )\\ {\dot{X}}_{2}=X_{2}∙(F(P)-D)\\ {\dot{X}}_{3}=X_{3}∙((1-Q(X_{1}+X_{3}))∙F_{2}(P)-D)+\alpha ∙X_{1}. \end{array}$$

These models contain an additional state variable, an evolved mutant, *X*_3_, which enters the system from its respective parent at a rate α. Its growth rate, *F*_*2*_*(P)*, is higher than that of the ancestral strain, *F(P)*, through an increase in μ_max_.

We fit these new models to our experimental data allowing for variation in the new parameters α and μ_max_ with all other parameters set to the best-fit value identified in the Results. We explored values of α within the range of 1x10^-10^ to 1x10^-4^, consistent with previously estimated mutation rates in non-mutator and mutator strains [61, 62]. We did not restrict μ_max_ since faster growth can be caused by changes in many different traits such as the loss of a costly function, increased nutrient uptake and metabolism, or cell size required for reproduction.

## Results

### Cultivation in a new growth medium that requires quorum sensing

As a first step, we formulated and tested a new growth medium suitable for our purposes. In several previous studies [21, 23, 30, 41, 46, 51, 54, 63], we and others have used the skim milk protein casein as a growth substrate that requires QS dependent proteolysis. Casein, in the form of the soluble salt caseinate, is an inexpensive and efficient protein source for *P*. *aeruginosa*, but it produces insoluble aggregates during culture growth. This property makes it difficult to measure cell density in real time using light scattering (OD_600_). To solve this problem, we considered gelatin as an alternative protein source.

**Fig 3:**
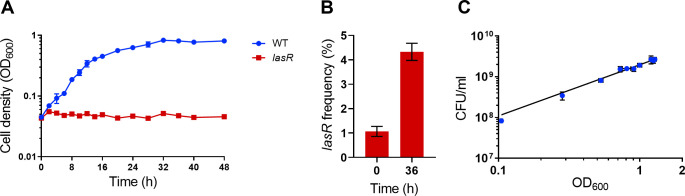
Batch culture growth in gelatin medium. (A) Cell densities (OD_*600*_) of *P*. *aeruginosa* WT (blue) and *lasR* mutant (red) monocultures grown independently in gelatin medium for 48 hours. (B) Initial and final *lasR* mutant frequencies after 36 hours of coculture growth in gelatin medium, where the WT and *lasR* mutants are grown together. (C) Correlation of cell densities (OD_*600*_) and CFU/ml of *P*. *aeruginosa* WT monoculture grown independently in gelatin medium batch culture. Each point is the average of three replicates with error bars showing standard deviation. In some cases, the standard deviation is too small to be seen.

We first evaluated the ability of *P*. *aeruginosa* to grow in minimal salts medium containing 1% gelatin as the sole carbon source (Fig 3). When grown independently, the WT exhibited growth saturating at an average OD_600_ of 0.8, whereas the *lasR* mutant maintained an average OD_600_ close to the initial inoculum of 0.05 (Fig 3A). This outcome shows that gelatin is a growth substrate that requires QS. During WT monoculture growth, the medium remained without microscopically visible precipitate and OD_600_ values correlated well with CFU/mL (Fig 3C, R^2^ = 93%). When initiated at a frequency of 1.0%, the *lasR* mutant strain enriched to a final frequency of 4.3% (Fig 3B), resulting in an average relative fitness of 1.4. These findings confirmed that the *lasR* mutant strain qualifies as a social cheater under our new growth conditions.

### Stability of the WT in the chemostat

**Fig 4:**
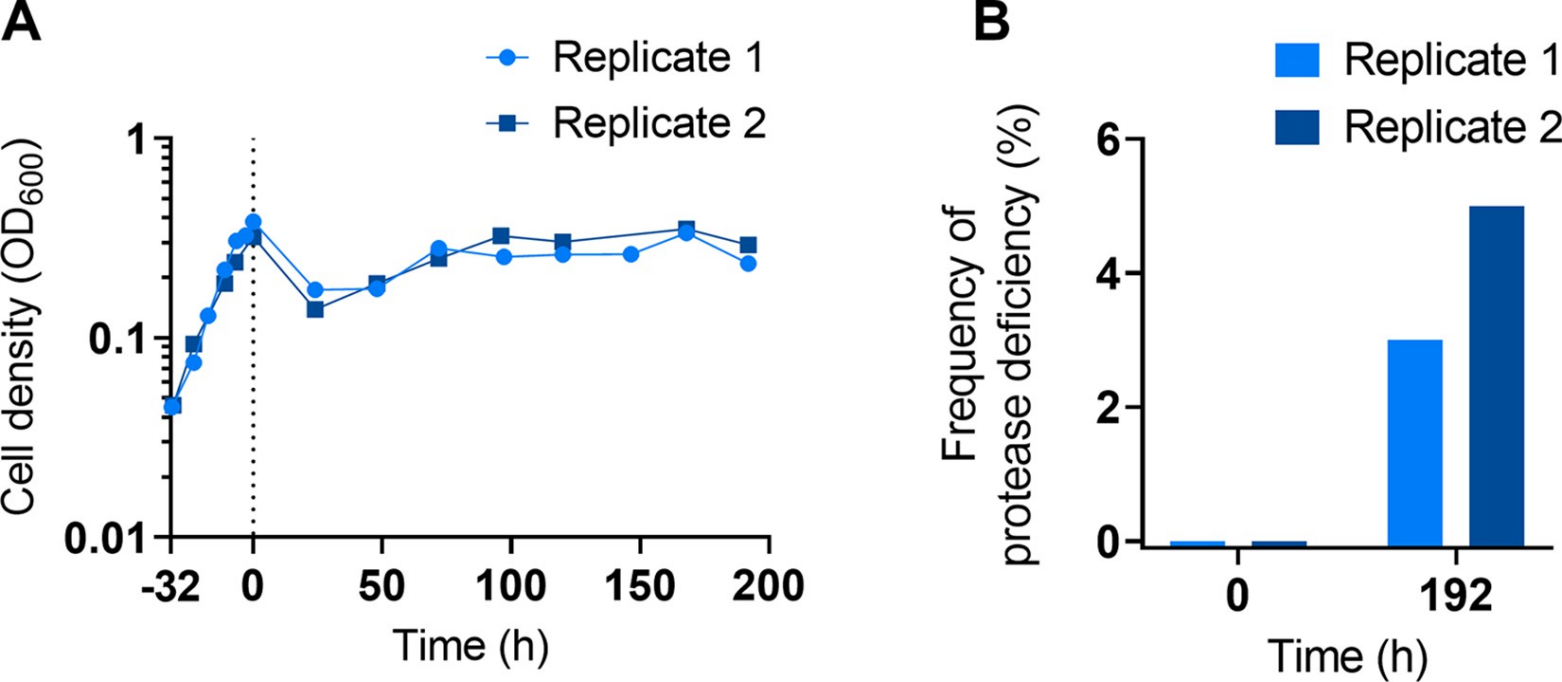
Stability of WT *P*. *aeruginosa* in the chemostat. A WT-only culture was grown in the chemostat over a period of almost 200 hours (9 days). The cultures were initially inoculated to a starting OD_*600*_ of approximately 0.05 and were grown in batch mode for approximately 32 hours. Then chemostat mode was initiated (indicated by the dotted line) and samples were taken approximately every 24 hours for 8 days. Each value is the measurement of a single replicate. (A) Total cell density (OD_*600*_). (B) Frequency of protease-deficient mutants at the first and last time points of chemostat mode as determined by the skim milk assay.

Next, we established growth conditions in the chemostat. Before initiating cocultures, we needed to demonstrate that the WT alone can achieve steady-state growth with gelatin as substrate. Upon inoculation of the chemostat, we allowed the culture to grow to late exponential phase before initiating flow. The dilution rate was then set to *D* = 0.12 1/h, a value that was below the growth rate achieved during the exponential phase in batch culture (μ = 0.17 1/h). At steady state, this dilution rate equates to a bacterial doubling time of 5.8 h. In two separate trials, the cell density transiently dropped after the flow was turned on but then stabilized at an OD_600_ of approximately 0.30 (Fig 4A), thus illustrating that a cooperator-only population is stable in the chemostat.

Samples of both trials were also screened for protease-deficiency using a skim milk assay to establish a baseline for our coculture studies below. Upon initiation of chemostat mode, both trials showed 0% protease-deficient colonies. By the end of the experiment, a small number of protease-deficient mutants had evolved with 3% in one replicate and 5% in the other (Fig 4B).

### Tragedy of the commons in the chemostat

**Fig 5:**
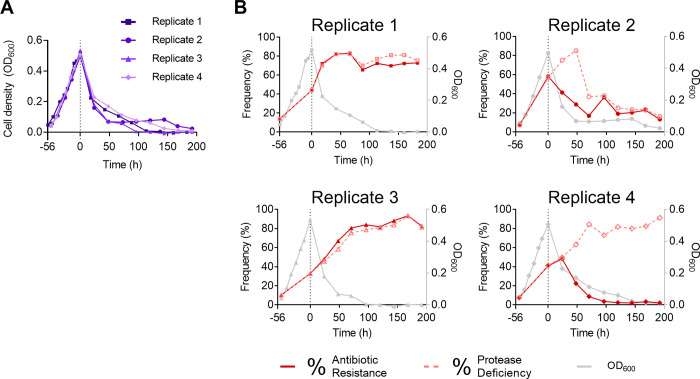
Cheater frequencies in chemostat replicates. Four replicates of a WT and *lasR* mutant coculture were grown in a chemostat over an approximately 10-day period with the first approx. 55 hours grown in batch mode and the latter approx. 192 hours (8 days) grown in chemostat mode (transition indicated by the vertical dotted line). The cultures were inoculated to a starting OD_*600*_ of approximately 0.05, at a 10% *lasR* mutant frequency. Samples were taken approximately every 24 hours for 8 days. (A) Total cell density (OD_*600*_) of the chemostat cocultures, plotted here on a linear scale to include cell density values of 0. (B) Each coculture replicate is graphed independently. Frequency of protease-deficient mutants in the chemostat cocultures as determined by skim milk assay (dashed lines) and frequency of *lasR* mutants as determined by antibiotic medium (solid lines). Cell density (OD_*600*_) from (A) (grey) is included for comparison.

We finally set out to determine if social cheating causes a Tragedy of the Commons in the chemostat by initiating cocultures of the WT and *lasR* mutant. The cocultures were inoculated to an initial *lasR* mutant frequency of 10% (rather than 1% in batch culture) for two primary reasons: (i) to ensure the burden on population growth caused by the *lasR* mutants could be observed within the experimental timeframe and (ii) to reduce the likelihood of other mutation events dominating the dynamics during a longer cocultivation period. When this WT/*lasR* mutant coculture was grown in the chemostat, a population collapse, or a Collapsing Tragedy, was indeed observed. Total cell density, as measured by OD_600_, increased significantly during batch mode, but steadily declined during chemostat mode to an OD_600_ below detection limit for three out of four replicates, and to approximately 0.05 for the remaining replicate (Replicate 2) (Fig 5A).

In addition to the total population dynamics, we determined the frequency of the defined, antibiotic-resistant *lasR* mutant using an antibiotic plate assay. We also assessed the frequency of protease-deficient mutants overall using our skim milk assay. This approach allowed us to discern if protease-deficient mutant cheaters other than the defined *lasR* mutant had evolved throughout the duration of the experiment. These protease-deficient mutants might include spontaneous *lasR* mutants evolved from the WT subpopulation, or *lasR-*independent mutants with loss-of-function mutations in genes encoding extracellular proteases, protease secretion machinery, or other regulatory proteins. This would make the defined *lasR* mutant cells a subpopulation of the protease-deficient population.

In batch mode, *lasR* and protease-deficient mutants increased concurrently in all cocultures. In chemostat mode, both frequencies remained congruent with one another in the first and third replicates, but differed in the second and fourth replicates, indicative of different mutational events. Conceivably, in the second replicate, a protease-deficient but antibiotic-sensitive mutant evolved that was eventually eliminated from the population, whereas in the final replicate, another protease-deficient but antibiotic-sensitive mutant evolved that ultimately eliminated the original defined *lasR* mutant (Fig 5B).

The general trajectories of protease-deficient mutants between the four coculture replicates also varied. In three of the replicates the frequency of protease-deficiency increased to approximately 80% before leveling out. This general behavior is congruent with model predictions (see below), albeit at a higher frequency. This higher than predicted frequency of protease-deficient mutants may suggest a mutation occurred in the *lasR* mutants that increased their relative fitness. Intriguingly, in the second Replicate the frequency of cheater mutants diminished. We investigated potential causes for these trajectories in a later section.

### Model parameterization and fit to experimental data

We had recently formulated a general mathematical model of the Tragedy in the Commons in a chemostat that predicted the stability of a WT-only population and a population collapse in a coculture setting under reasonable conditions [15]. Our data generally support this predicted outcome; however, we wanted to investigate whether the model would show those same outcomes when fitted with biologically relevant parameters specific to our study. This would determine whether the model captures all the essential mechanisms of the system and would allow us to test its predictive efficacy. We formulated and parameterized a deterministic ODE chemostat model as described in Methods, with the concentration of cooperator and cheater strains, as well as public good enzyme, growth substrate, and product as the relevant state variables. We performed numerical simulations of this model in comparison with the experimental results from the previous sections.

To estimate the initial variable concentrations and parameter values that best represent our experiment, we utilized data from our own experiments as much as possible. For example, the initial concentration of bacteria, the dilution rate, and the substrate concentration are all determined by experimental design. We also estimated the cost of QS-dependent cooperation from experimental data. Other parameters, including those that are challenging to measure, are taken from the literature (Table 1; see *S1 File* for a full explanation of each parameter derivation). While some parameters are conclusively one fixed value (e.g. the dilution rate as determined by experimental design), others were identified to be within a possible biological range (e.g. the rate of enzyme-substrate turnover and the initial enzyme concentration as taken from literature sources). Although not every combination of parameters within these estimated ranges results in dynamics which mirror our experimental data, we nonetheless find many which do (*S1 File*).

**Table 1:** Variable and parameter values used for model simulations.

Variable or parameter	Units	Biological meaning	Initial condition or parameter range^§^	Best fit value
*S*	mmol/l	Substrate (gelatin) concentration	0.200 ≤ *S*(0), *S*^*0*^ ≤ 0.250	0.210
*X*_*1*_	g dry weight/l	Cell density of the *P*. *aeruginosa* WT strain	WT-only: *X*_*1*_(0) = 0.0240	0.024
			Coculture: *X*_*1*_(0) = 0.0216	0.0216
*X*_*2*_	g dry weight/l	Cell density of the *P*. *aeruginosa lasR* strain	Coculture: *X*_*2*_(0) = 0.00240	0.00240
*E*	mmol/l	Enzyme (protease) concentration	0.0 < *E*(0) ≤ 2.424x10^-5^	3.00x10^-6^
*P*	mmol/l	Product (amino acid) concentration	P(0) = 0.0	0.00
*D*	1/h	Dilution rate	0.121	0.121
μ_max_	1/h	Maximal growth rate of *P*. *aeruginosa*	1.38	1.38^¶^
*k*_*cat*_	1/h	Rate of enzyme-substrate turnover	40 ≤ *k*_*cat*_ ≤500	480
*K*_*S*_	mmol/l	Amount of product (P) at half maximal growth	0.840	0.840
*K*_*M*_	mmol/l	Gelatin concentration at half maximal enzyme reaction rate	0.0580 ≤ *K*_*M*_ ≤ 0.0725	0.068
η	mmol/g dry weight	Enzyme produced per bacterial biomass	0.0 < η ≤ 126x10^-5^	620 x 10^−6^
γ	g dry weight/mmol	Product-dependent growth yield conversion of nutrient to biomass	0.0423	0.0423
σ	N/A	Number of product molecules produced per substrate molecule	36.0 ≤ σ ≤ 46.0	40.0
*q*	N/A	Metabolic burden of enzyme production at high cell density	0.0425 ≤ *q* ≤ 0.625	0.610
*QS*_*min*_	g dry weight/l	Density of cooperative WT cells needed for QS to begin	0.0447 ≤ *QS*_*min*_ ≤ 0.161	0.045
*n*	N/A	Hill coefficient determines speed of transition from little to maximum *q*	*n* ≥ 2.00	2.00

§ In some cases, the literature provided a range of values for a variable or parameter. Rather than taking the average, we explore the full range.

¶ The μ_max_ value chosen here is larger than what we calculated for batch phase growth in gelatin medium, because it is based on product rather than substrate utilization kinetics. The full explanation can be found in Supporting Information (S1 File_.

**Fig 6:**
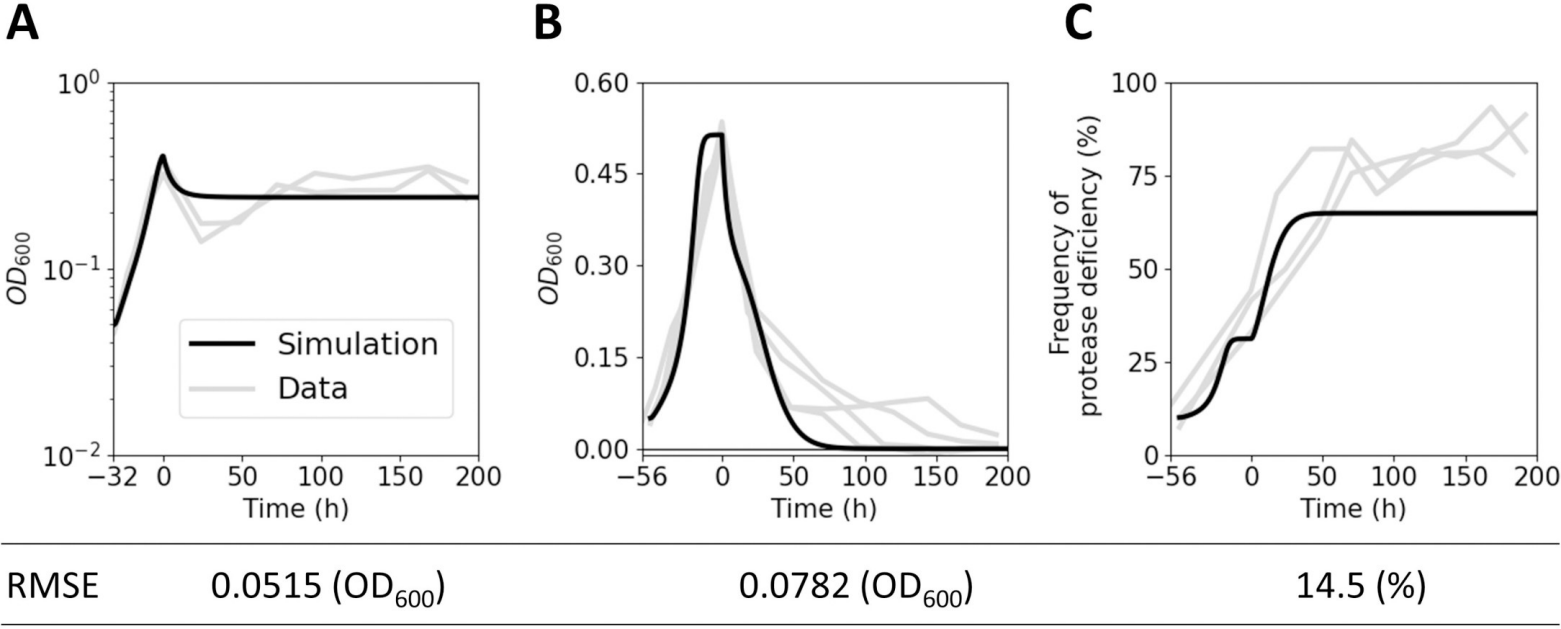
Simulated best fit. The combined best fit of the ODE model (black) to all three experimental data sets (grey) is shown: WT-only OD_*600*_ (A), coculture OD_*600*_ (B), and coculture protease-deficiency (C). The corresponding parameter values are as described in Table 1.

To identify the parameter combination that most closely matches our experimental results, we fit the model to three experimental data sets: WT-only total density (Fig 6A), coculture total density (Fig 6B), and cheater frequency as determined by protease-deficiency (Fig 6C). For the first two, we considered the entire data set, and for the latter, we only considered the three replicates with congruent protease-deficient trajectories (coculture replicates 1, 3 and 4)—disregarding the second replicate with a much lower and decreasing protease-deficient frequency. We considered the protease-deficient mutant frequency in the following because it represents total proportion of cheaters that impose a metabolic burden on cooperative growth. We used a least-squares analysis to fit our mathematical model to all three data sets simultaneously (Fig 6), choosing an overall best fit that minimized the combined root mean square error. Table 1 shows the variables and parameter values that generated the combined best fit to all three data sets and were then used for the numerical simulations and analysis below—unless indicated otherwise.

While the *lasR* and protease-deficient cheater frequencies differed between experimental coculture replicates, it is worth noting that each of these frequencies appeared to eventually stabilize. We can indeed show mathematically that the *lasR* cheater frequency will always approach a constant limit, meaning that the value will not change over time. In fact, the cheater frequency from the model

$$R=\frac{X_{2}}{X_{1}+X_{2}}$$

would be expected to saturate to

$$R(t)=\frac{R(0)}{1-R(0)+R(0)e^{\int_{0}^{t}\;A(t)dt}}e^{\int_{0}^{t}\;A(t)dt},$$

where $A(t)=Q(X_{1}(t))F(P(t))$. Thus, as *t* approaches infinity, *R*(*t*) approaches some constant value, *c*, such that 0 < *c* ≤ 1 (see S3 File for the full mathematical proof). Thus, while the population collapses, both cooperators and cheaters remain present. Cheaters do not reach fixation as the competitive exclusion principle would predict (S3 File). The reason for this is that as cheaters invade, the cooperator density eventually falls below the QS threshold (*QS*_*min*_) such that the effective cost of QS (*Q*(*X*_*1*_)) tends to zero. With a negligible cost to QS, there is no longer an observable selective advantage for cheaters, resulting in the coexistence of cooperators and cheaters at nearly identical growth rates. Because QS is density-dependent, the limit at which the cheater frequency saturates as the population collapses is density-dependent.

### Analysis of model sensitivity to individual parameters

**Fig 7:**
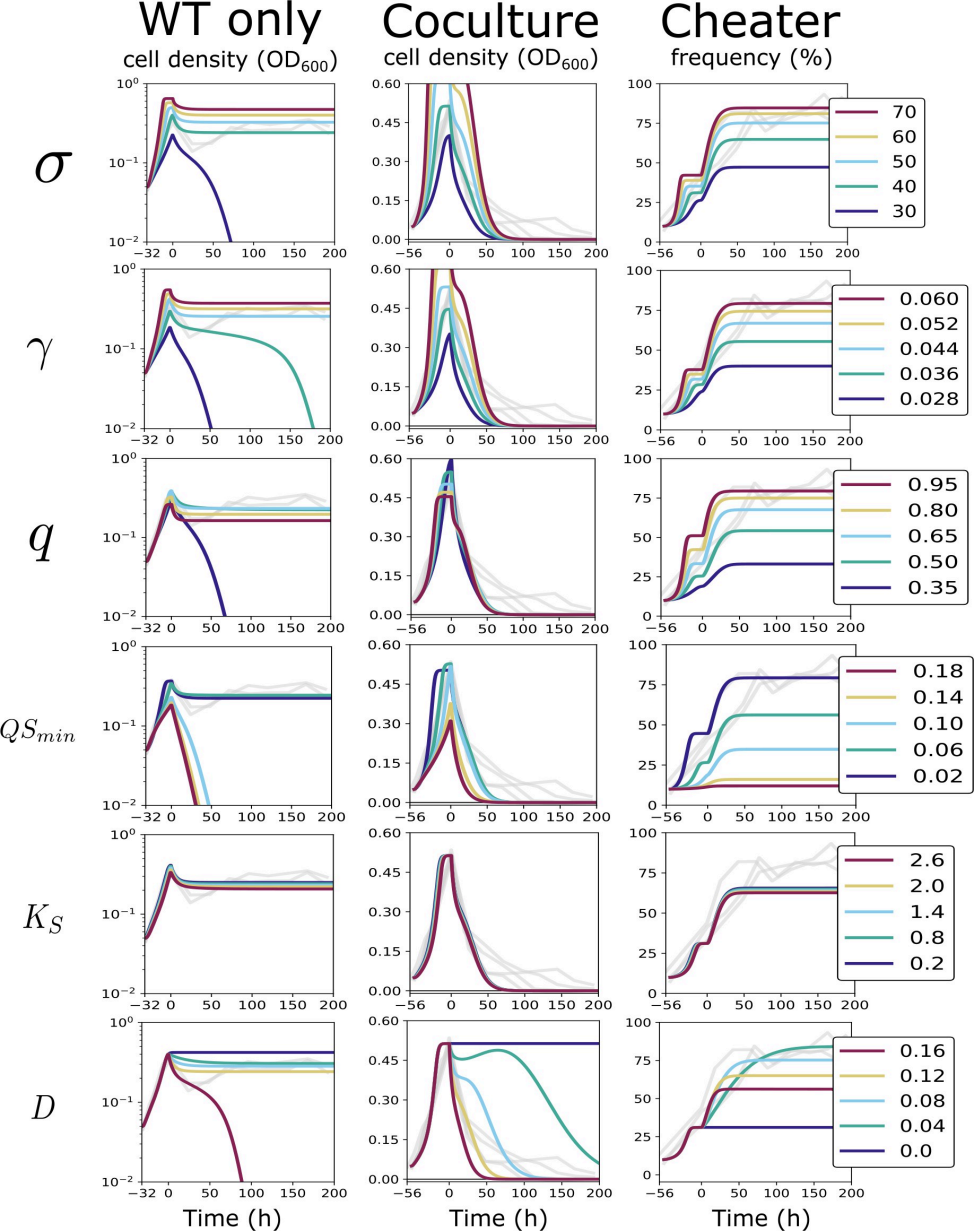
Parameter sensitivity analysis. Simulations were run to illustrate the impact of changing a single parameter on the system dynamics. Rows indicate the parameters from top to bottom as follows: number of products produced per substrate molecule (*σ*), nutrient to biomass conversion (*γ*), metabolic burden of enzyme production (*q*), minimum cooperator cell density needed for quorum sensing to begin (*QS*_*min*_), the concentration of product at half-maximal growth (*K*_*S*_), and the dilution rate (*D*). The columns indicate the WT-only (OD_*600*_), coculture (OD_*600*_), and cheater frequency (%) scenarios from left to right. The experimental data previously described (Figs 4 and 5) are shown in grey while the simulations are shown in color with dark blue being the lowest simulated value and red the highest simulated value. All other parameters besides the one identified by the row heading remain as specified in Table 1. In places where all five lines are not clearly visible, they are overlapping.

Beyond data fitting, our mathematical model shows how changes in biological parameters and initial conditions impact the dynamics of the cell densities and cheater frequencies. Given many of our parameters are estimated, we also explore these parameters outside of their estimated ranges to capture a greater view of the model dynamics. The number of product molecules produced per substrate molecule (*σ*) and the product to biomass conversion factor (γ) are highly influential to the system impacting the batch phase growth, long-term stability of WT-only culture, and the *lasR* cheater frequency limit. In contrast, the estimated parameter ranges of others, such as the amount of product at half-maximal growth (*K*_*S*_), is sufficiently narrow such that those parameters have little impact on the system dynamics. Parameter changes also varied in their effect on individual time courses. For example, the metabolic burden (*q)* affected the coculture and cheater frequency trajectories much more than the WT-only trajectory (Fig 7).

For many of the parameters, the effect on system dynamics emerges from their contribution to a given auxiliary function, i.e. the metabolic burden of enzyme production (*Q*(*X*_*1*_)), enzyme-substrate catalysis (*G*(*S*,*E*)), and bacterial growth (*F*(*P*)) functions. As such, the parameters within these functions can have inverse effects on the population dynamics. For example, decreasing *QS*_*min*_ or increasing *q* will both increase the total cheater frequency in the population (Fig 7).

Interestingly, regardless of the initial condition, the WT-only simulations resulted in either stability or washout, whereas the coculture simulations always resulted in washout as a consequence of cheater invasion, representing a Collapsing Tragedy of the Commons, unless there is no dilution (Fig 7). Hence, continuously diluted chemostat cocultures are predicted to always cause population collapse, in contrast to undiluted batch cultures. This illustrates that a positive dilution is a requirement for the Collapsing Tragedy to occur. However, too high of a dilution can cause cooperator-only populations to collapse as well, and the inability for the cooperative WT-only populations to establish stability when the dilution is sufficiently high aligns with previous theory that has been mathematically proven for similar chemostat models [15, 34]. See S4 File for simulations illustrating the impact on the system dynamics for all parameters.

Given the way single parameter modifications can have opposing impacts on the system, we further explored the combined effects of select parameter pairs by analyzing the average RMSE obtained from model fits to each data set individually. We chose parameters within the QS function (*q* and *QS*_*min*_), *σ*, and *γ* for this analysis because the model’s stability of the WT-only population density was relatively sensitive to changes in these values.

**Fig 8:**
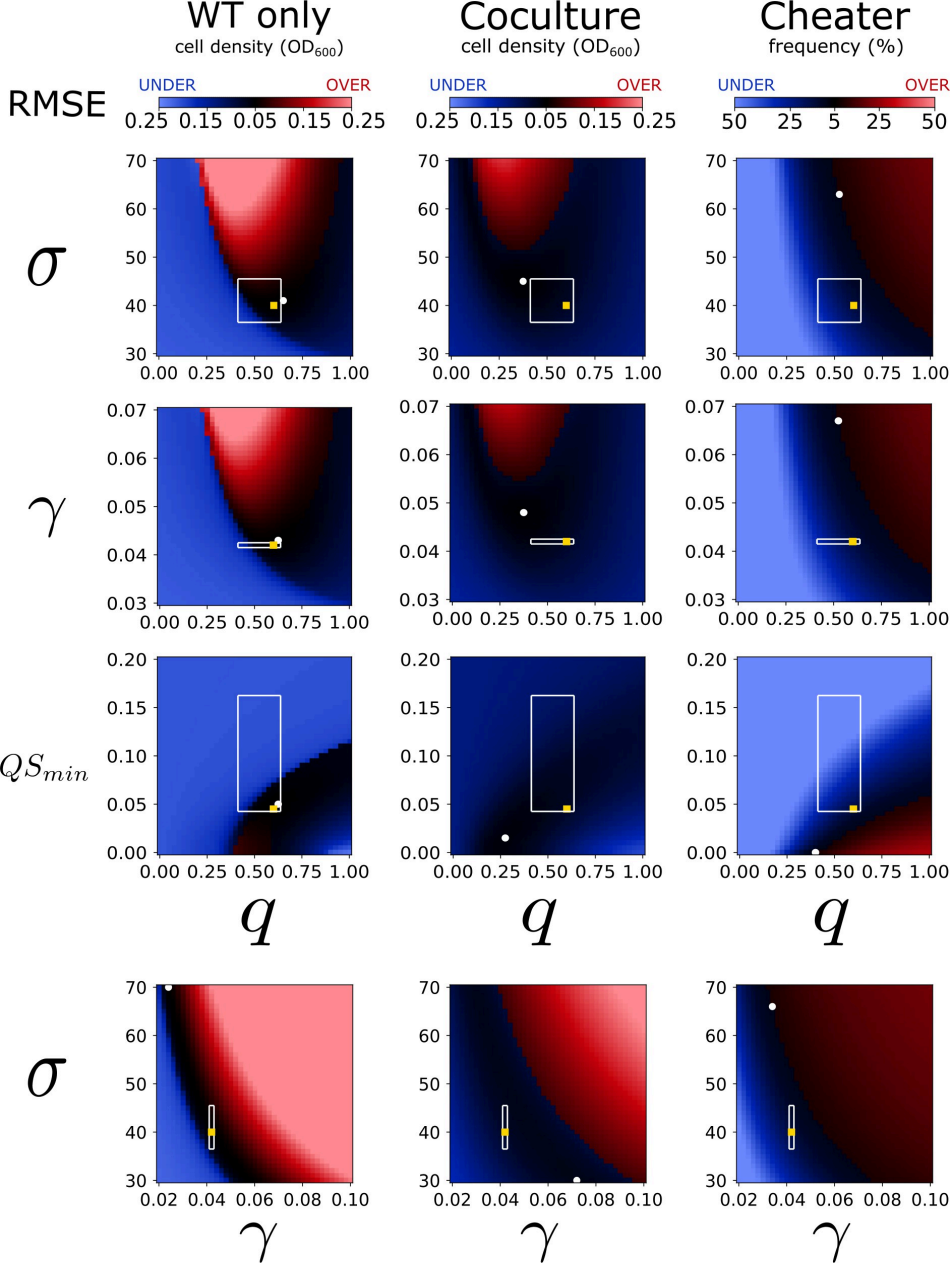
RMSE heat map for variable pairs with extended ranges. Heatmaps indicating the average RMSE across an extended range of four parameters in four pairwise combinations. The RMSE is shown in red where the simulated values were greater than the experimental data ("overshoot”) and is shown in blue where the simulated values were below the experimental data (“undershoot”). In both cases a darker color indicates a lower RMSE. The four variables and the ranges simulated were the number of enzymatic cleavage products (*σ*; [30,70]), metabolic burden (*q;* [0,1]), nutrient to biomass conversion (*γ*; [0.03,0.07]), and the minimum cooperator cell density needed for quorum sensing to begin (*QS*_*min*_; [0,0.2]). The pairs from top to bottom are: *σ* and *q*, *γ* and q, *QS*_*min*_ and *q*, and *σ* and *γ*. A white circle indicates the lowest RMSE and a gold square indicates the best fit value. Each parameter range was divided into 41 equidistant values for simulation. The white lines run along the edges of the simulated values that were the closest to the maximum and minimum estimated value as described in Table 1. The simulations in Fig 7 illustrate the dynamics across one row or column of these heat maps.

Using the RMSE map to infer how the dynamics of the system change in response to variable pairs shows increasing *σ* or *γ* impacts the dynamics in similar ways (Fig 8). *q* and *QS*_*min*_ have a similar but opposite relationship as *σ* and *γ*. Increasing *q* has the same effect as decreasing *QS*_*min*_ (Figs 7 and 8) which is an expected result given the relationship these variables have to the QS function (Fig 2). The congruent impact of σ and γ is not as obvious and may not have been as easily discovered without this analysis.

Notably, these extended parameter best fit values are not much lower than the within-range fit values; the maximum difference between the combined best fit RMSE (Fig 6) and the extended range individual data best fits are 0.003 (OD_600_) for the WT-only culture, 0.0248 (OD_600_) for the coculture, and 6.0 (%) for the cheater frequency data (see S5 File). Although there are other parameter choices which may return a sufficiently or similarly good fit, this analysis underscores the accuracy of the model since the model makes predictions which align with the data from relevant biological parameter estimates.

These RMSE maps can also be used to indirectly illustrate the behavior of the system. Our experimental data for the WT-only cultures showed stable growth, so a high RMSE (bad fit) indicates either undershooting (population collapse) or overshooting. Our experimental data for the co-cultures show a population collapse, so a high RMSE can only indicate that the model has overshot the data prior to collapsing. For example, increasing σ causes an overshoot effect and reducing it can cause a washout of the WT (compare the top and bottom left panels of Fig 8 in a vertical direction to the top left panel of Fig 7). We used divergent colors to illustrate when the simulation values were, on average, either over or under the data. For the WT only data, a sufficiently high RMSE which undershoots the data indicates a population collapse and can be interpreted from Fig 8 as a solid-colored light blue zone. This further underscores that a minimum threshold for the metabolic burden, *q*, is needed to achieve stability, and finds that threshold to be approximately 0.25 for these parameter pairs—although the true threshold could be higher.

Taken together, this analysis shows that cooperating populations can only sustain themselves at steady-state levels within a narrow range of parameters and initial conditions. We also show that lower values of the metabolic burden *q* decreases the relative fitness of cheaters, and that a minimal threshold of *q* is necessary to sustain steady-state growth of the WT (Figs 7 and 8). Finally, we illustrate that continuous dilution is a requirement to observe a population collapse (Fig 6).

### Resolving discrepancies in cheater frequency

Our model simulations primarily deviated from the experimental results with respect to the cheater mutant frequency (Fig 6). One possible explanation for these discrepancies is that distinct mutations occurred in each replicate population during extended chemostat cultivation. This hypothesis is supported by the emergence of a small number of cheaters in the WT-only chemostat culture (Fig 5A) and by strikingly divergent frequencies for protease-deficient and antibiotic-resistant *lasR* mutant subpopulations in one chemostat coculture replicate (Fig 4B; Replicate 4). Thus, guided by our mathematical model, we sought to better understand the underlying mutations that may have occurred and their impact on population dynamics. Inspired by previous work that illustrates the capacity for faster-growing mutants to evolve [23, 52], we explored the possibility of similar mutations evolving in our chemostat cultures (herein referred to as “evolved mutants”; Table 2).

**Table 2:** Phenotypes of defined and evolved *P*. *aeruginosa* strains.

Strain or mutant		Enzyme production^†^	Antibiotic resistance†	Faster growth^‡^
D E F I N E D	WT	✓	–	–
	*lasR* (mini-Tn7-Tp)	–	✓	–
E V O L V E D	Cheater from *lasR*	–	✓	✓
	Cheater from WT	–	–	✓
	Cooperator from WT	✓	–	✓

† Enzyme production and antibiotic resistance was determined by differential plating on skim milk or antibiotic plates, respectively.

‡ Faster growth, relative to the defined strains, was determined by comparing the relative fitness of evolved cheaters against the defined *lasR* cheater and through numerical modeling predictions

In Replicates 1 and 3, the frequencies of protease-deficient and antibiotic-resistant *lasR* mutants were very similar, however, these cheaters occurred at a frequency higher than what the model predicts. This discrepancy could point to a mutation emerging from the *lasR* mutant lineage that permits faster growth. In Replicate 4, the frequency of protease-deficiency was greater than that of antibiotic-resistance, which, if caused by a mutation, would suggest a faster-growing cheater evolved, potentially from an antibiotic-sensitive WT parent. It is also possible that a *lasR* mutant lost its antibiotic resistance, although we believe this to be a highly unlikely occurrence for several reasons, including (i) the antibiotic resistance is encoded in a neutral chromosomal site, rather than a plasmid, and is highly stable over at least 100 generations [47], (ii) there are no appreciable differences in the growth rates between strains with or without the antibiotic marker [46], and (iii) there is no selection for or against antibiotic resistance in the chemostat.

In Replicate 2, mutant frequencies initially mirrored those of Replicate 4, except that the evolved cheaters (also likely from an antibiotic-sensitive WT parent) were unable to maintain their fitness advantage and eventually realigned with the *lasR* mutants at a low frequency. The low frequency of protease-deficient and *lasR* mutant cheaters in Replicate 2 could in turn be due to the late evolution of a faster-growing cooperator.

**Fig 9:**
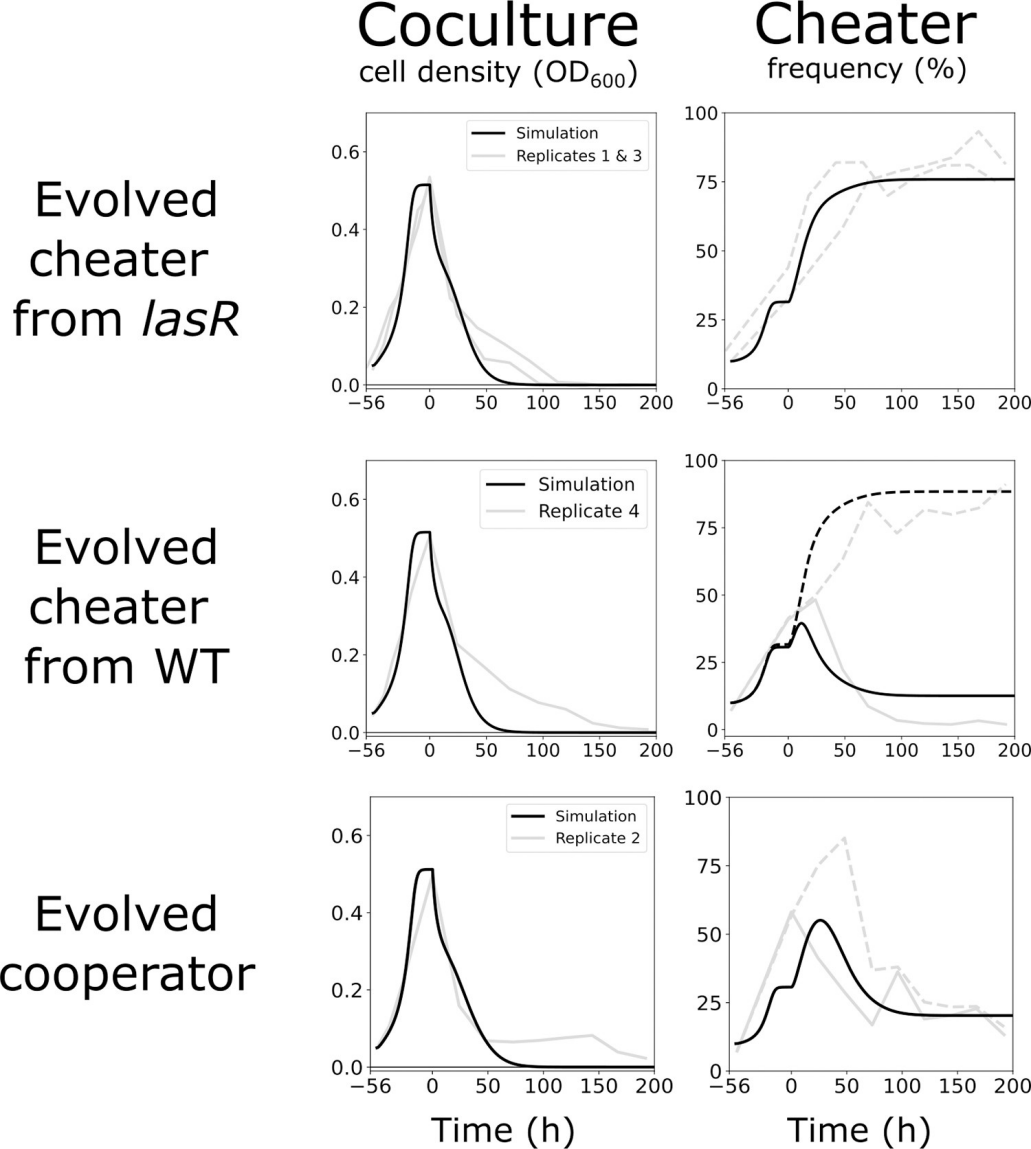
Simulated effect of evolved cheaters on coculture dynamics. Model simulated with three different faster-growing evolved mutants from top to bottom as follows: (i) cheater evolving from a *lasR* mutant parent, (ii) cheater evolving from a WT parent, and (iii) a cooperator evolving from a WT parent. The model numbers (i–iii) correspond to the model numbers in the Methods section. The left column shows the total cell density, and the right column is the cheater frequency. The model simulation is shown in black, and the experimental data previously described (Figs 4 and 5) are shown in grey with Replicates 1 and 3 on the top, Replicate 4 in the middle, and Replicate 2 on the bottom. In the cheater frequency graphs, dashed lines show the protease-deficient subpopulation, and solid lines indicate the antibiotic-resistant subpopulation. All simulated values are the same as in Table 1 except the added mutation rate (α) and the faster growth (μ_*max*_) of the evolved mutants. The respective α and μ_*max*_ values for each replicate are as follows: 1x10^*-4*^ and 3.8 for the evolved cheater from *lasR*, 7x10^*-7*^, 4.0 for the evolved cheater from WT, and 3x10^*-5*^ and 4.0 for the evolved cooperator.

We first explored these hypotheses numerically, by incorporating the mutational events into our mathematical model to simulate the effects of evolved mutants on population dynamics. We considered a faster-growing protease-deficient mutant evolving either from a *lasR* mutant ancestor (Replicates 1 and 3) or from a WT parent that does not have antibiotic resistance (Replicate 4). A faster-growing protease-deficient mutant from a WT parent could result from two consecutive mutations, however, it could result from a single mutation as well. For example, a mutation in a global regulator can impact multiple behaviors and/or processes at once. For simplicity, we only consider the case where one mutation has occurred in our model. We also considered a faster-growing cooperator mutant (Replicate 2). Although the situation is likely more complex for Replicate 2 and the evolved cooperator is not the only mutant, we include this analysis as a proof of concept to illustrate that such a mutation could result in a reduced *lasR* mutant cheater frequency without increasing overall cell density. The introduction of these mutants yields cheater frequency trajectories that are representative of our experimental data (Fig 9). The addition of a faster-growing cheater evolving from a *lasR* mutant ancestor results in a higher frequency of protease-deficient cheaters as seen in Replicates 1 and 3. The introduction of a faster-growing cheater evolving from a WT ancestor maintains a high frequency of protease-deficient cheaters and, at the same time, greatly reduces the frequency of the antibiotic-resistant *lasR* mutant cheater. A faster-growing cooperator can reduce the cheater frequency without increasing population density (Fig 9). Simply incorporating one mutant fails to fully capture the dynamics of that replicate, which is not surprising as there are likely additional mutations causing the rise and fall of protease-deficient and antibiotic-sensitive mutants throughout.

Since the existence of these mutations is speculative at this point, we sought experimental validation and empirically investigated their presence in chemostat Replicates 1, 3, and 4. We reasoned that in each of these cases, divergent trajectories could be explained by a single mutational event that can be captured by our screening and cultivation methods.

We screened for the existence of faster-growing cheaters by taking three protease-deficient isolates from the final time point of coculture Replicates 1, 3, and 4. We took antibiotic-resistant isolates from Replicates 1 and 3, because we predict those mutants evolved from a defined *lasR* mutant ancestor. In contrast, we selected antibiotic-sensitive isolates from Replicate 4, because we predicted evolution from a WT, and therefore antibiotic-sensitive, ancestor. We then compared the relative fitness of each isolate to that of the defined *lasR* mutant control, when grown together with the WT in a gelatin medium coculture.

**Fig 10:**
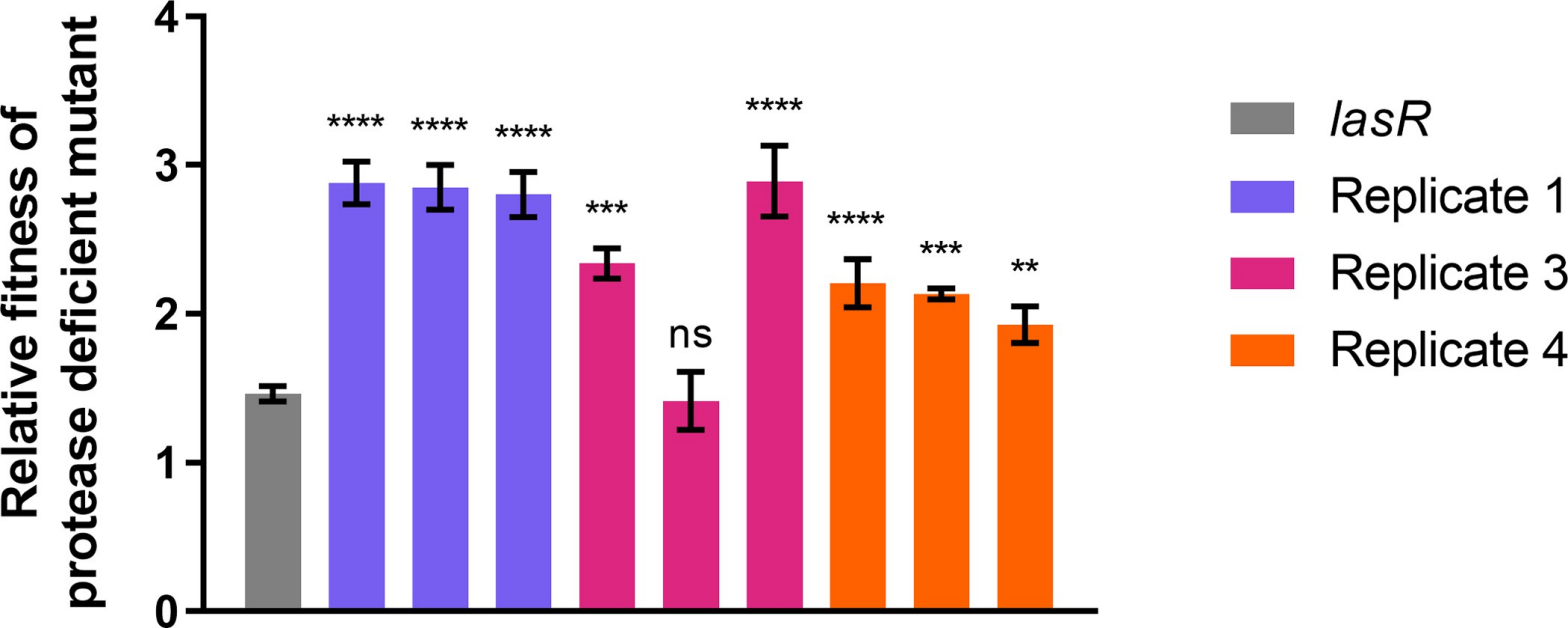
Relative fitness of the evolved and defined protease-deficient mutants. The average relative fitness after 36 hours of growth with the WT of the defined *lasR* mutant (grey) and three evolved mutants (protease-deficient isolates) taken from the final chemostat timepoint of Replicates one (blue), three (magenta), and four (orange). All evolved cheaters had a significantly higher relative fitness as determined by a one-way ANOVA between each replicate and the *lasR* control with an *ad hoc* Bonferroni correction, with one exception in the third Replicate (*P* values are indicated above each bar in the conventional manner as follows: *P* > 0.05 ns, *P* ≤ 0.05 *, *P* ≤ 0.01 **, *P* ≤ 0.001 ***, and *P* ≤ 0.0001 ****. Specific *P* values can be found in Supporting Information (*S5 File*: Table S5.2).

All three evolved isolates from Replicates 1 and 4, and two out of three replicates from Replicate 3 showed a relative fitness significantly above that of the defined *lasR* mutant (Fig 10). These results also suggest that evolved mutants rise to high frequency in each chemostat population by the end of the cultivation period. It is likely that the one non-significant isolate from Replicate 3 was simply an ancestral *lasR* mutant cheater because both the emergent and *lasR* mutants are protease-deficient and antibiotic-resistant. Taken together, this analysis provides experimental evidence that chemostat cultivation under conditions that favor cooperation selects for faster-growing cheater adaptations.

## Discussion

The purpose of the paper was two-fold: first, to determine if the Collapsing Tragedy occurs in the chemostat according to model predictions and second, to investigate the predictive capabilities of such models. Using a new growth medium suitable for chemostat cultivation, we first established that *P*. *aeruginosa* is capable of cooperative proteolytic growth in a chemostat system, that the *lasR* mutant strains not contributing to protease production are unable to grow on their own, and that the *lasR* mutants have a fitness advantage when mixed with the WT cooperator in a coculture. These three properties define social cheating in a population and they can ultimately lead to a population collapse [3, 5].

We then established if the population-level dynamics of the chemostat match the general mathematical theory of microbial social behavior [15]: we observed the stability of the WT population alone and a large decline in population density when a *lasR* mutant is introduced. Generally, all replicate populations experienced a Tragedy of the Commons, defined as a substantial reduction in group fitness as a consequence of selfish behavior–with or without a complete collapse (Rankin et al. 2007). Strictly speaking, only three of the four replicates experienced a Collapsing Tragedy defined as a complete population collapse. Replicate 2 maintained a very low population density at the end of the cultivation period, presumably as a result of specific mutation events.

Although we observed the theoretically predicted outcome, we had also considered that coexistence or cooperator dominance could be an outcome of the experiment. Cooperation could have been promoted by the beneficial cooperator mutations we speculated to have occurred in coculture Replicate 2 or by cheater control mechanisms such as punishment and metabolic constraint [25, 26, 30, 64]. Punishment of cheaters via cyanide or pyocyanin production has been reported as a control mechanism against social cheaters in *P*. *aeruginosa* [25, 26], although this effect is context-dependent [48]. The experiments that found these toxic extracellular substances to be an effective form of cheater control were conducted in batch culture with a serial transfer regime, and this methodological difference could have allowed more of the substance to build up whereas the chemostat would dilute it. Thus, dilution may play an important role in the efficacy of such mechanisms. The co-regulation of both public and private nutrient acquisition by LasR has also been shown to restrict *lasR* cheaters in populations of *P*. *aeruginosa*, however, it requires the presence of specific substrates that are metabolized intracellularly in a QS-dependent fashion [30]. Our experiment did not include any such substrates, although a mutation which privatized the public good or carbon source we used could also potentially explain the cheater frequency dynamics of Replicate 2. In principle, these processes could have been occurring during our experiment; however, they would have been included in our calculations for their relative growth rates and would not have been modeled explicitly.

It is also possible that the initial cheater frequency of our populations was simply too high, allowing cheater invasion to levels that promote population collapse [24] before beneficial cooperator mutations have a chance to rise to a sufficiently high frequency, and rendering mechanisms that would mitigate cheating at low frequencies ineffective. Another reason we may have observed a collapse instead of coexistence maintained by some biological mechanism is that our microbial population was well-mixed, eliminating population structure and spatial effects as factors that favor cooperation [2, 3, 5, 53, 65–67].

Despite the growth advantage the cheaters had, they did not fix in the population. This seemingly contradicts expectations; however, we showed mathematically that the final cheater frequency is density-dependent due to QS. This is because the cooperator population seizes to produce enzyme when its density decreases below the quorum threshold, thereby eliminating the growth advantage the cheaters had. This illustrates that both total population density and the cheater frequency throughout cultivation play a vital role in determining the final cheater frequency, similar to findings from another study [28].

For population density data, a good fit between the model and experiment was achieved within established parameter ranges, supporting our claim that the model captures the essential mechanisms of cooperation and cheating via enzyme production as a public good in *P*. *aeruginosa*. Such parametrization is important for quantifying the costs and benefits of cooperative behavior and defining the boundary conditions within which cooperation can occur and remain stable. The metabolic burden of cooperating (*q*) has a central role here. Investment into cooperation must be sufficiently high to produce enough enzymes to enable proteolytic growth, but increased investment in turn means increased vulnerability to cheating.

Further, we established that a positive dilution rate is a requirement for the Collapsing Tragedy to occur, suggesting that there must be a sufficient turnover of nutrients and the population. This could have implications in the use of social microbes to treat bacterial infections without antibiotics [68]. Treatment might be particularly effective in environments with high fluid flow, such as the urinary tract. The treatment of other types of infections, such as topical wounds, would require regular washing to eliminate the infection.

In contrast to the population-level data, the phenotypes of subpopulations differed between experimental replicates in cocultures and could not be accurately captured with a single parameter set. In this case, a modified model that incorporates mutational events which may have occurred (Fig 9) combined with experimental validation of the existence of such mutations (Fig 10) was able to resolve these differences. The divergence between our antibiotic resistance and skim milk proteolysis data underscores the importance of directly measuring both the phenotype of interest and the initial strain during long term cultivation, when possible. Had we only measured skim milk proteolysis, the mutation in coculture Replicate 4 would not have been identified. We concluded that cheater mutants evolved from defined *lasR* mutant and WT strains in Replicates 1,3 and 4, respectively. Despite the occurrence of these evolved mutants, it is unlikely that they are the driver of the Collapsing Tragedy that ensues as soon as flow is initiated. Mutations occur at very low frequencies and take time to build in the population. Our simulations further illustrate that a Collapsing tragedy is inevitable even in the absence of evolved cheaters, in a coculture solely comprised of defined cheaters and the WT (Fig 6).

A faster-growing protease-proficient mutant, as we predict occurred in Replicate 2, averted a Collapsing Tragedy but still did not fully recover population growth. The near-collapse of that population suggests that while the protease-proficient mutant grew faster than the parent WT and *lasR* mutant, its growth was not fast enough to enrich in the chemostat. This is likely due to the continued dilution of the chemostat environment maintaining a low cell density and thus limiting QS-dependent enzyme induction. The population density of Replicate 2 fell below the best-fit value for the QS threshold (*QS*_*min*_ at an OD_600_ of 0.094; Table 1 and S1 File) on the second day of chemostat mode and remained below that threshold for the remainder of the experiment. Essentially, faster-growing cooperators are *capable* of producing protease, but they are being kept below the QS threshold by constant dilution and are therefore not actively producing the enzyme.

Most populations experience multiple selection pressures, and the chemostat is no different. Additional adaptations beyond what we explored may have contributed to the observed variability of the *lasR* and protease-deficient cheater frequencies. It is also conceivable that some of the variation is caused randomly by genetic drift as populations reach low cell densities during the wash-out phase. However, we believe the mutant phenotypes we observed are the result of natural selection because (i) population numbers are relatively high for much of the growth phase (still approx. 100 million cells in the chemostat vessel when OD_600_ values are near the detection limit of about 0.001; Figs 3C and 4B), (ii) mutants with very similar behavioral phenotypes were found in three of the replicates at relatively high frequency, and (iii) there is evidence for adaptation to a cooperative growth environment from other studies [23, 24, 69]. Additional modeling and experimentation, such as sequencing the genomes of the evolved mutants or adding stochasticity to the dilution rate to examine the role of genetic drift and bottlenecking, would be a compelling direction for future projects.

Using the chemostat culturing system to assess the validity and predictive capabilities of theory against empirical results has additional benefits, as the chemostat has been considered an ideal laboratory system for studying populations due to its properties reflecting natural ecosystems in many respects [53, 70, 71]. It has continuous inputs and outputs of nutrients analogous to the natural turnover of resources in nature. Population densities often reach a steady state in the chemostat, balancing growth with dilution to maintain an unchanging concentration; the latter being akin to the death, predation, and emigration that would occur in natural ecosystems. Many natural ecosystems resemble the chemostat dynamics, and chemostat models have been applied to the understanding of the human gut [72] and the flow of organic matter in rivers [73, 74]. Establishing the limitations of these models is then crucial as they begin to be used to make predictions about human health and the environment. Thus, the utilization of the chemostat environment to investigate cooperation offers ecologically relevant insights into the maintenance of cooperation in microbes and, potentially, beyond.

Taken together, the results of this study underscore three main ideas. The first is the importance of validating theoretical models with experimental outcomes, when possible. Doing so allows us to identify the extent to which we can utilize theoretical results to predict biological outcomes and phenomena. In our case, the model we developed accurately predicted population density outcomes, but was unable to capture the variable subpopulation dynamics of cheater mutant frequencies. Had we relied solely on the results of the theoretical predictions to understand the outcome of social cheating in the chemostat environment, we would have missed the selection pressure the environment has on creating newly evolved phenotypic variants.

Secondly, we illustrate the multiple benefits of integrating empirical data into the theoretical framework. Such an integration of empirical data and mechanistic modeling revealed insights into the underlying mechanisms that are most influential on the system dynamics. Another benefit of creating a biologically accurate model tuned to a specific study system is that it can then be used to explore possible outcomes and generate testable predictions. The subpopulation dynamics we observed were variable and unable to be captured by a mechanistic model, but we were able to use our model as a tool to generate hypotheses about the root of those discrepancies by incorporating mutations and, in turn, experimentally validated their existence. By using the model to identify mutations that explain our data we were able to reduce the amount of empirical work needed to identify what, if any, mutations had occurred. The need for integration between theoretical and empirical work is further supported by the fact that such an integration is responsible for the creation of some of the more successful branches of social evolution theory, such as sex-ratio evolution theory [4].

Lastly, this study illuminates how even a seemingly simple experimental system and biological process is challenging to fully encapsulate deterministically—especially when given sufficient time for evolution to occur. Although additional integration of mechanistic modeling and empirical work is still needed, the results of this study suggest that incorporating stochastic effects may be necessary for understanding and predicting evolutionary outcomes. Notably, the chemostat environment coupled with competition from social cheaters created a selection force resulting in phenotypic evolution throughout the course of the experiment. Such adaptation has been observed in previous *in vitro* evolution experiments with social microbes [23, 24, 69]. The evolution of these new traits supports the notion that the Tragedy of the Commons may not be a gravestone signaling the end of a species, but may instead be a stepping stone to a different community structure containing a new dominant species [75, 76].

## Supporting information

S1 File Model parameterization. (PDF)

S2 File Proof the mathematical model is well-posed. (PDF)

S3 File Poof of cheater frequency saturation. (PDF)

S4 File Sensitivity analysis of all parameters. (PDF)

S5 File Statistical analysis tables. (PDF)
