# Supplementary material for: Putting theory to the test: An integrated computational/experimental chemostat model of the tragedy of the commons
Source: PLoS One. 2024 Apr 10;19(4):e0300887. doi: 10.1371/journal.pone.0300887 (PMC11006152; doi:10.1371/journal.pone.0300887)
Supplement: S1 File — (PDF) [file pone.0300887.s001.pdf]

S1. Model parameterization

Initial concentration of gelatin in the chemostat, $S(0)$:

Our M9 gelatin medium contains 1 g of gelatin per 100 ml, or equivalently 10 g per liter.

The strength, or stiffness, of gelatin is indicated by its bloom value. The brand of gelatin used is Alfa Aesar Type A with a bloom of 175, and the average molecular weight of gelatin with a bloom of 175 to 225 is in the range 40 to 50 g/mmol according to product information sheet on www.sigmaaldrich.com.

We can estimate $S(0)$ using the average molecular weight and quantity of gelatin. For the case where we estimate the average molecular weight as 50 g/mmol, we calculate this as follows:

$$S(0) = \frac{1 \text{ mmol}}{50 \text{ g}} \cdot \frac{10\text{g}}{\text{l}} = 0.20 \frac{\text{mmol}}{\text{l}}.$$

Since the average molecular weight of gelatin is estimated to be between 40 and 50 g/mmol, we get

$$0.20 \frac{\text{mmol}}{\text{l}} \leq S(0) \leq 0.25 \frac{\text{mmol}}{\text{l}}.$$

Initial concentration of *P. aeruginosa* strains in the chemostat, $X_1(0)$ and $X_2(0)$:

Sexton *et al.* determined that an OD_{600} =1 is equivalent to 0.48 grams of dry weight per liter for *P. aeruginosa* grown in a chemostat in minimal medium [1].

By inoculating the bioreactor at an OD_{600} =0.05 we can determine the initial population density in g DW/l (either $X_1(0)$ alone or both $X_1(0)$ and $X_2(0)$ in coculture).

For the wild type only culture:

$$X_1(0) = 0.05 \cdot 0.48 \frac{\text{gDW}}{\text{l}} = 0.024$$

For the coculture:

$$X_1(0) = 0.05 \cdot 0.9 \cdot 0.48 \frac{\text{gDW}}{\text{l}} = 0.022$$

$$X_2(0) = 0.05 \cdot 0.1 \cdot 0.48 \frac{\text{gDW}}{\text{l}} = 0.0024$$

Initial concentration of enzyme in the chemostat, *E(0)*:

P. aeruginosa produces a number of different endo and exoproteases that contribute to protein degradation [2]. For simplicity, we focus here on the best-characterized endoprotease, LasB elastase, which is known to play a major role in the degradation of protein substrates such as gelatin [3]. The average concentration of LasB elastase in the supernatants of elastase-producing *P. aeruginosa* in LB stationary phase cultures is 30.90 µg/ml [4].

Given that the molecular weight of the LasB protein is 33 g/mmol [5,6] we can determine the average concentration as

$$E(0) = \frac{0.0309\text{g}}{\text{l}} \cdot \frac{1\text{ mmol}}{33\text{ g}} = 9.36\text{x}10^{-4}\,\frac{\text{mmol}}{\text{l}}.$$

The range of enzyme measured in that study was 0-80 µg/ml, with zero representing values that were below the detectable limit. Thus, the actual initial condition could lie anywhere between

$$0.0\,\frac{\text{mmol}}{\text{l}} < E(0) \leq 2.42x10^{-3}\,\frac{\text{mmol}}{\text{l}}.$$

This range represents the amount of protease enzyme we expect to find in the batch culture prior to inoculation into the chemostat bioreactor. The bioreactor was inoculated from stationary-phase LB cultures to an OD_{600} of 0.05, resulting in a 100-fold dilution. We consider the same dilution factor for mono and cocultures, neglecting the small reduction in enzyme concentration through inoculation with 90% of the WT strain in cocultures. Thus, the amount of enzyme in the bioreactor will be

$$0.0\,\frac{\text{mmol}}{\text{l}} < E(0) \leq 2.42\text{x}10^{-5}\,\frac{\text{mmol}}{\text{l}}.$$

Initial concentration of product in the chemostat, *P*(0):

We refer to amino acids produced by extracellular protease as “products” collectively. In the experimental design, the chemostat is inoculated from LB stationary phase cultures. We assume that all the usable nutrient and product has been exhausted by the organism in the stationary phase cultures. This means that no product, or possibly a negligible amount of product, is being transferred.

Furthermore, proteolysis-deficient *lasR* mutants show little to no growth in the 1% gelatin medium (Figure 3 of main text) indicating that there are no breakdown products in fresh gelatin medium. Thus,

$$P(0) = 0.$$

Chemostat dilution rate, *D*:

The equation to convert peristaltic pump speed in revolutions per minute (RPM) to flow rate was determined experimentally by measuring the time it took to fill a beaker with 10 ml of water at four different pump speeds: 5, 15, 35, and 65 RPM (Table S1).

Table S1.1: Duration to achieve specific volume at various pump speeds.

RPM	time (min) to fill 10 ml	ml/min
5	30	0.33
15	10	1.0
35	4.0	2.5
65	2.1	4.8

Using linear regression, we were then able to determine the following equation to convert pump speed (x) to flow rate (y) as $y = 0.0731x$ (Figure S1).

The chemostat experiment is operated at an RPM=2.75 with a bioreactor volume of 100 ml. The resulting flow rate is determined by

$$y = 2.75\,\text{RPM} \cdot 0.0731\,\frac{\text{ml}}{\text{min} \cdot \text{RPM}} = 0.201\,\frac{\text{ml}}{\text{min}} = 12.1\,\frac{\text{ml}}{\text{h}},$$

and the flow rate is then converted to our dilution rate (D) is given by

$$D = 12.1\,\frac{\text{ml}}{\text{h}} \cdot \frac{1}{100\text{ ml}} = 0.121\,\frac{1}{\text{h}}.$$

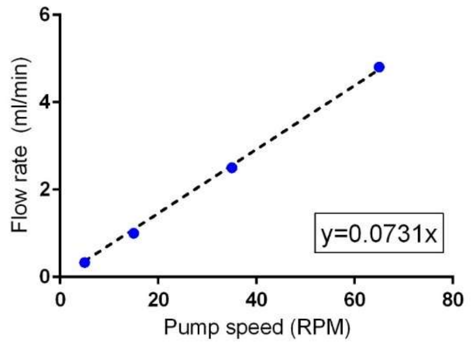

Fig S1.1. Correlation between pump speed and flow rate. Linear regression was used to establish an equation which converts pump speed (RPM) (x) to flow rate (ml/min) (y).

Maximum growth rate constant, $\mu_{\mathbf{max}}$:

The growth rate, *F(P)*, models the growth of *P. aeruginosa* as a Monod equation dependent on product concentration (*P*). As indicated above, the products are primarily individual amino acids that are the result of extracellular proteolysis. μ_{max} is estimated to be 1.38 1/h when grown in minimal medium containing a 1% mixture of amino acids (casamino-acid, CAA) [7].

Of note, we calculated an exponential-phase growth rate of $\mu = 0.17$ from our batch cultures. That calculation is based on substrate degradation (*S*). Since our system of equations models growth from the product of degraded substrate (i.e. we use *F(P)* instead of *F(S)*), it is more accurate to use the growth rate observed from CAA consumption.

Rate of enzyme-substrate turnover, k_{cat}:

A lower bound for the rate of enzyme-substrate turnover was estimated to be 1 mass % *S* / (mass % *E* · min) [7], where mass % *S* and mass % *E* are the biomass concentrations (in w/v) of the substrate and enzyme respectively. 1 mass % equals 1 g of E or S per 100 ml of solution, or 10 g per l.

Here, we convert mass % to molar concentrations (in mmol/l). For *S* we get $S = \frac{10\text{ g}}{\text{l}} \cdot 0.02\frac{\text{mmol}}{\text{g}}$, where we consider the molecular weight of gelatin to be 50 g/mmol.

The molar concentration of $E = \frac{10\text{ g}}{\text{l}} \cdot \frac{1\text{ mmol}}{33\text{ g}}$ using the molecular weight of LasB protein to be 33 g/mmol [5,6].

Converting this to k_{cat} we get:

$$1\frac{\text{mass }\%\, S}{\text{mass }\%\, E\ \cdot\ \text{min}} = \frac{10\text{ g/l}\ \cdot\ 0.02\text{ mmol/g}}{10\text{ g/l}\ \cdot\ 0.03\text{ mmol/g}\cdot\text{min}} \cdot \frac{60\text{ min}}{\text{h}} = 40\ 1/\text{h}$$

Given the molecular weight of gelatin was estimated to be between 40 and 50 g/mmol (see above), this yields an estimation of:

$$40 \leq k_{cat} \leq 50.$$

The authors suggest that the conditions of their experiment likely underestimated k_{cat}, and that the true k_{cat} rate is higher. Indeed, many catalysis rates for the enzymatic activity of proteases lie in the hundreds and thousands per hour [8,9], and other enzymes produced by *P. aeruginosa* have a catalysis rates in the thousands to the tens or hundreds of thousands per hour [10,11]. For these reasons, we expanded the upper bound of the range approx. 10-fold to get

$$40\frac{1}{\text{h}} \leq k_{cat} \leq 500\frac{1}{\text{h}}$$

Amount of product present at half-maximal growth rate, K_s:

K_s is challenging to measure. One reason for this is the difficulty of measuring very low concentrations of growth-limiting product at steady-state [7,12]. However, in *P. aeruginosa,* the K_s for CAA has been estimated as 0.01 mass %, or equivalently $\frac{0.01\text{ g}}{100\text{ g}}$, from data fitting [7]. We first convert this estimate into g/l as follows:

$$\frac{0.01\text{ g}}{100\text{ g}} = \frac{0.01\text{ g}}{0.1\ l} = 0.1\frac{\text{g}}{\text{l}}$$

The average molar mass of amino acids is 118.885 g/mol = 0.119 g/mmol [13].

$$K_s = 0.1\frac{\text{g}}{\text{l}} \cdot \frac{1}{0.119}\frac{\text{mmol}}{\text{g}} = 0.840\frac{\text{mmol}}{\text{l}}$$

Substrate concentration at half-maximal enzyme reaction rate, K_{M}:

K_{M} is approximated as 0.29 mass %, or equivalently $\frac{0.29\text{ g}}{100\text{ ml}} = \frac{2.9\text{ g}}{\text{l}}$ [7].
The brand of gelatin used is Alfa Aesar Type A with a bloom of 175, and the average molecular weight of this kind of gelatin is 40 to 50 g/mmol.

$$\frac{2.9\text{ g}}{\text{l}} \cdot \frac{\text{mmol}}{50\text{ g}} \leq K_M \leq \frac{2.9\text{ g}}{\text{l}} \cdot \frac{\text{mmol}}{40\text{ g}}$$

$$0.058\frac{\text{mmol}}{\text{l}} \leq K_M \leq 0.0725\frac{\text{mmol}}{\text{l}}$$

Amount of enzyme produced per bacterial biomass, η:

The concentration of LasB elastase in an LB stationary-phase culture has a range of 0-80 μg/ml (where 0 represents falling below a detectable limit), with an average of 30.9 μg/ml (equivalently 0.0309 g/l) [4] and a growth yield of OD_{600} between 4 and 5.

An OD_{600} between 4 and 5 is equivalent to 1.92 and 2.40 gDW/l respectively [1].

The molar mass of elastase is 33,000 g/mol = 33.0 g/mmol [5,6]. The amount of LasB per bacterial biomass with an OD_{600} of 4 is then calculated to be

$$\eta = \frac{1\text{ l}}{1.92\text{ gDW}} \cdot 0.0309\ \frac{\text{g}}{\text{l}} \cdot \frac{1\text{ mmol}}{33.0\text{ g}} = 0.000488\ \frac{\text{mmol}}{\text{gDW}}.$$

If we consider the range for an OD_{600} between 4 and 5, we get $0.000390 \leq \eta \leq 0.000488$. However, while the average concentration was 30.90 μg/ml, the range is 0-80 μg/ml - further increasing the uncertainty to

$$0.0\ < \eta \leq 0.00126$$

Nutrient uptake to biomass conversion factor, γ:

We consider amino acids to be the main products of gelatin proteolysis, which entails LasB and other endo- and exopeptidases [2]. In minimal medium containing 0.5% (w/v), or 5 g/l, casamino acids, *P. aeruginosa* grows to an OD_{600} of approximately 3.7 [14]. We can calculate population density in gDW/ml from the OD_{600} [1] as follows: 3.7·0.48 g/l = 1.776 g/l.

The average molar mass of amino acids is 118.89 g/mol = 0.119 g/mmol [13]. Thus, the growth yield is:

$$\gamma = 1.776\ \frac{\mathrm{g}}{\mathrm{l}} \cdot \frac{1\ \mathrm{l}}{5\ \mathrm{g}} \cdot 0.119\ \frac{\mathrm{g}}{\mathrm{mmol}} = 0.0423\ \frac{\mathrm{g}}{\mathrm{mmol}}.$$

Number of product molecules produced per substrate molecule in enzyme-substrate catalysis, σ:

The brand of gelatin used (Alfa Aesar Type A) has a bloom of 175 and an average molecular weight of 40 to 50 g/mmol.

The average molar mass of amino acids is 118.885 g/mol = 0.119 g/mmol [13].

The number of amino acids produced, if all of the substrate was converted into usable products, is in the range of $\frac{40\ \mathrm{g/mmol}}{0.119\ \mathrm{g/mmol}}$ to $\frac{50\ \mathrm{g/mmol}}{0.119\ \mathrm{g/mmol}}$, or 336 to 420 respectively.

However, when comparing growth yields in M9 gelatin and in M9 CAA media, it is apparent that only a fraction of the gelatin is converted to usable product. The average growth yield of wild type *P. aeruginosa* with 1% (w/v) gelatin was OD_{600} = 0.808 (Figure 3 of main text), whereas the average growth yield with 0.5% casamino acids is OD_{600} = 3.7 [14].

Thus, the conversion factor of usable product is $\frac{10\,\frac{\mathrm{g}}{\mathrm{l}}}{5\,\frac{\mathrm{g}}{\mathrm{l}}} \cdot \frac{3.7}{0.808} = 9.16$.

Therefore, $\frac{336}{9.16} \leq \sigma \leq \frac{420}{9.16}$ or equivalently $36.7 \leq \sigma \leq 45.9$.

Since the breakdown product molecules can only exist as whole units (i.e. half of an amino acid cannot be produced), we extend this parameter range slightly to ignore fractions yielding:

$$36 \leq \sigma \leq 46.$$

Metabolic burden of enzyme production as a fraction of the total growth rate, q:

We can determine q from the ratio of growth rates between strains (also termed relative fitness) during the batch phase of chemostat cultivation. However, we know from previous work that the *lasR* mutant does not enrich at a constant rate during coculture growth [14] and hence the WT does not experience a constant metabolic burden of enzyme production. In fact, following inoculation and prior to reaching a quorum, the *lasR* mutant initially grows at the same rates as the WT in coculture, presumably because both strains benefit from the products present in the

inoculum. These include secreted proteases and proteolysis products carried over from the pre-culture. We can attempt to more accurately determine relative fitness by disregarding this initial phase and only considering the phase of *lasR* mutant enrichment.

Based on this previous work [14], the phases of no enrichment vs. enrichment can be distinguished by the total cell density, with a density of approx. 2.5-4x10^8 CFU/ml indicating the initial point of *lasR* mutant enrichment. This point overlaps with the QS threshold determined below. We can then estimate the initial *lasR* mutant cell densities at this point according to the mutant fraction quantified upon inoculation of our chemostat cocultures, and we can determine the final cell densities directly from our measurements at the end of the batch phase of our chemostat cocultures.

First, we consider the case where the initial total estimated CFU/ml at point of *lasR* mutant enrichment is 2.5x10^8. In this case, the first and second chemostat coculture replicates have a *lasR* mutant density of 3.43x10^7 and 1.83x10^7 respectively. After 49 hours in batch mode, the first replicate reached a total CFU/ml of 9.67x10^8 and a *lasR* mutant CFU/ml on antibiotic medium of 2.70x10^8. The second replicate ended batch mode after 55.5 hours with a total CFU/ml of 1.98x10^9 and a *lasR* mutant CFU/ml of 7.17x10^8.

Thus, for the first replicate we can calculate the absolute fitness of the WT and *lasR* strains as:

$$\text{WT abs fit} = \ln\left(\frac{9.67\text{x}10^8 - 2.70x10^8}{2.5x10^8 - 3.43x10^7}\right) = 1.17$$

$$\text{lasR abs fit} = \ln\left(\frac{2.7x10^8}{3.43x10^7}\right)$$

Hence the relative fitness of the *lasR* mutant is 2.06/1.17=1.76.

Following a similar calculation as above for the second replicate, we can determine the absolute fitness of the WT and the *lasR* mutant fitness to be 1.70 and 3.66, respectively.

For the second replicate, the relative fitness of the *lasR* mutant is 3.66/1.70=2.15.

We calculate the reduced growth of the WT, and *q,* by setting the *lasR* mutant growth rate equal to 1, or 100% of the possible growth. For the first replicate we get:

$$\begin{aligned} 1-q &= \frac{1}{1.76} = 0.568 \\ q &= 0.432. \end{aligned}$$

Similarly, considering the second replicate where the relative fitness is 2.15, we get $q = 0.535$.

Repeating the steps above for an initial total CFU/ml of 4x10^8, the first and second replicates will have a *lasR* density of 5.48x10^7 and 2.92x10^7, respectively. Then reproducing the above calculations with these new initial densities yields a relative fitness of 2.29 and 2.60 for Replicate 1 and Replicate 2, respectively. Thus, for Replicate 1 *q*=0.563 and for Replicate 2 *q*=0.615.

Hence, we will consider q to be within the range of the minimum and maximum estimated q:

$$0.432 \leq q \leq 0.615.$$

However, to simplify the numerical analysis we consider:

$$0.425 \leq q \leq 0.625.$$

Density of cooperators needed for quorum sensing, $QS_{\min}$:

It is estimated that the QS threshold occurs somewhere in the range of $1.0 \times 10^8 \leq$ CFU/mL $\leq 5.0 \times 10^8$ based on QS gene expression data [14]. The corresponding value in gDW/mL can be determined by first converting CFU/mL to OD_{600} according to the correlation in Fig. 1C, and then converting OD_{600} to gDW/mL using the conversion factor as before. The correlation in Fig. 1C yielded a best fit equation of

$$OD_{600} = 10^{\left(\frac{log(CFU/mL) - intercep}{slope}\right)}$$

where the slope is 1.031 to 1.482 and the intercept is 9.272 to 9.320. Considering the average slope and intercept, 1.257 and 9.296 respectively, CFU/mL values of 1×10^8 and 5×10^8 yield OD_{600} values of approx. 0.0931 and 0.335, respectively. We can then multiply the OD_{600} by the conversion factor 0.48 to obtain gDW/mL [1]. This gives us a range of

$$0.0447 \leq QS_{min} \leq 0.161.$$

Hill coefficient for QS function, n:

There is evidence that QS in *P. aeruginosa* functions like an 'on-off' switch, with positive autoregulation and receptor dimerization as the underlying mechanisms [15–17]. To obtain this type of behavior in our model with the QS function $Q(X_1)$, we require an ultrasensitive response with a Hill coefficient $n > 1$ [18,19]. A Hill coefficient of 2 is commonly used to model the rheostat like behavior of bacterial growth, thus, we use n=2 in this study.

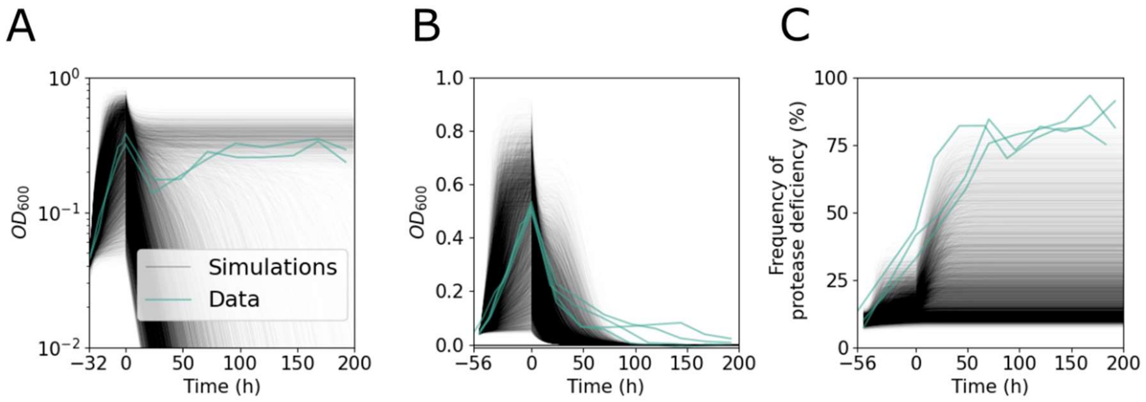

Fig S1.2. Line graphs illustrating 10,000 simulations with the estimated parameter and initial condition values randomly chosen from the estimated parameter ranges for three experimental data types: (A) WT-only population density, (B) coculture cell density, and (C) the cheater frequency. Experimental data is shown in green and simulations are shown in an opaque black line, thus, darker regions occur where more simulations overlap with one another. This figure shows that growth conditions mirroring our results are possible within our established estimated parameters.

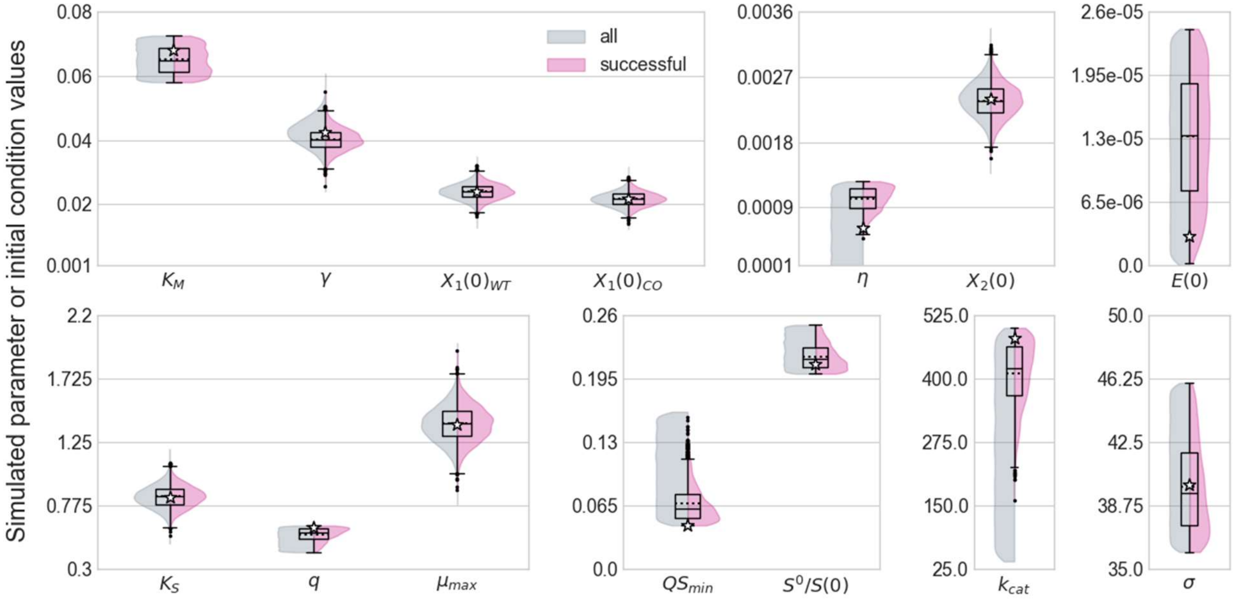

Fig S1.3. Box plot of 100,000 chemostat simulations where randomly chosen parameter and initial condition values produced successful simulations as determined by having a solution with a positive WT-only cell density and cheater frequency at the final time point and a coculture final batch mode density that is sufficiently low – under an OD_{600} of 0.58. 2454 of the simulations were successful. A violin plot of all simulated values is shown on the left (grey) and successful values shown on the right (red). Parameters and initial conditions that were initially estimated to be within a range were randomly chosen from a normal distribution, and those estimated to be a single value were selected from a normal distribution with the estimate as the mean (grey). The white star indicates our best-fit solution values. The initial conditions of the WT differed between the WT-only and coculture and are indicated as such by a subscript of "WT" and "CO" respectively.

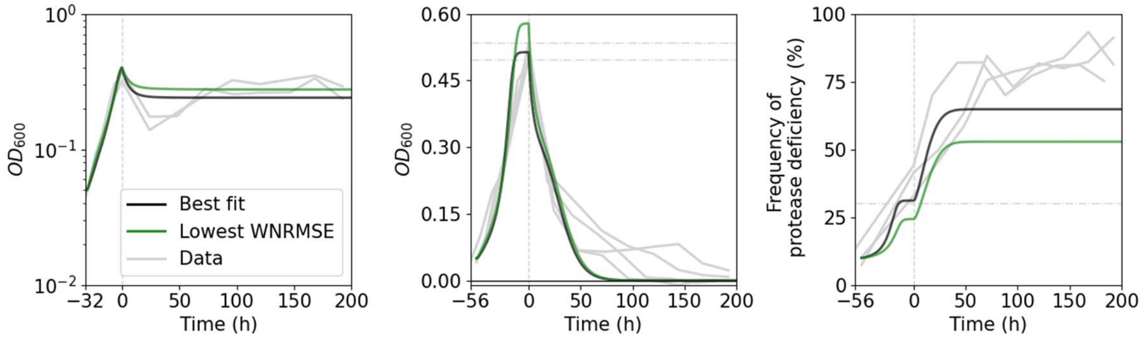

Fig S1.4. Comparison of the best fit as determined by the lowest weighted normalized RMSE values (WNRMSE) (green) and the final best fit (black) across three data sets: WT only (left), coculture (center), and cheater frequency (right). Solid light grey lines show the chemostat data for comparison. A vertical dashed grey line indicates the transition from batch mode to chemostat mode. Horizontal dashed grey lines indicate the range (coculture) or minimum (cheater frequency) cutoffs used to find the simulation with the overall best fit.

References

1. Sexton DJ, Schuster M. Nutrient limitation determines the fitness of cheaters in bacterial siderophore cooperation. Nat Commun. 2017;8: 230. doi:10.1038/s41467-017-00222-2

2. Robinson T, Smith P, Alberts ER, Colussi-Pelaez M, Schuster M. Cooperation and cheating through a secreted aminopeptidase in the *Pseudomonas aeruginosa* RpoS response. mBio. 2020;11. doi:10.1128/mBio.03090-19

3. Marquart ME, Caballero AR, Chomnawang M, Thibodeaux BA, Twining SS, O'Callaghan RJ. Identification of a novel secreted protease from *Pseudomonas aeruginosa* that causes corneal erosions. Invest Ophthalmol Vis Sci. 2005;46: 3761–3768. doi:10.1167/iovs.04-1483

4. Coin D, Louis D, Bernillon J, Guinand M, Wallach J. LasA, alkaline protease and elastase in clinical strains of *Pseudomonas aeruginosa*: quantification by immunochemical methods. FEMS Immunol Med Microbiol. 1997;18: 175–184. doi:10.1111/j.1574-695X.1997.tb01043.x

5. Bever RA, Iglewski BH. Molecular characterization and nucleotide sequence of the *Pseudomonas aeruginosa* elastase structural gene. J Bacteriol. 1988;170: 4309–4314. doi:10.1128/jb.170.9.4309-4314.1988

6. Kida Y, Higashimoto Y, Inoue H, Shimizu T, Kuwano K. A novel secreted protease from *Pseudomonas aeruginosa* activates NF-κB through protease-activated receptors. Cell Microbiol. 2008;10: 1491–1504. doi:10.1111/j.1462-5822.2008.01142.x

7. Cezairliyan B, Ausubel FM. Investment in secreted enzymes during nutrient-limited growth is utility dependent. Proc Natl Acad Sci. 2017;114: E7796–E7802. doi:10.1073/pnas.1708580114

8. Dall'Acqua W, Halin C, Rodrigues ML, Carter P. Elastase substrate specificity tailored through substrate-assisted catalysis and phage display. Protein Eng Des Sel. 1999;12: 981–987. doi:10.1093/protein/12.11.981

9. Turk BE, Huang LL, Piro ET, Cantley LC. Determination of protease cleavage site motifs using mixture-based oriented peptide libraries. Nat Biotechnol. 2001;19: 661–667. doi:10.1038/90273

10. Elliott BW, Cohen C. Isolation and characterization of a lysine-specific protease from Pseudomonas aeruginosa. J Biol Chem. 1986;261: 11259–11265. doi:10.1016/S0021-9258(18)67377-6

11. Knight DA, Finck-Barbançon V, Kulich SM, Barbieri JT. Functional domains of Pseudomonas aeruginosa exoenzyme S. Infect Immun. 1995;63: 3182–3186. doi:10.1128/iai.63.8.3182-3186.1995

12. Coleman KD, Fowler AC. A mathematical model of exoprotein production in bacteria. Math Med Biol J IMA. 1984;1: 77–94. doi:10.1093/imammb/1.1.77

13. Hachiya T, Terashima I, Noguchi K. Increase in respiratory cost at high growth temperature is attributed to high protein turnover cost in Petunia × hybrida petals. Plant Cell Environ. 2007;30: 1269–1283. doi:10.1111/j.1365-3040.2007.01701.x

14. Sandoz KM, Mitzimberg SM, Schuster M. Social cheating in *Pseudomonas aeruginosa* quorum sensing. Proc Natl Acad Sci. 2007;104: 15876–15881. doi:10.1073/pnas.0705653104

15. Scholz RL, Greenberg EP. Positive Autoregulation of an Acyl-Homoserine Lactone Quorum-Sensing Circuit Synchronizes the Population Response. mBio. 2017;8: e01079-17. doi:10.1128/mBio.01079-17

16. Smith P, Schuster M. Antiactivators prevent self-sensing in *Pseudomonas aeruginosa* quorum sensing. Proc Natl Acad Sci. 2022;119: e2201242119. doi:10.1073/pnas.2201242119

17. Schuster M, Li C, Smith P, Kuttler C. Parameters, architecture and emergent properties of the Pseudomonas aeruginosa LasI/LasR quorum-sensing circuit. J R Soc Interface. 2023;20: 20220825. doi:10.1098/rsif.2022.0825

18. Goryachev AB. Understanding bacterial cell−cell communication with computational modeling. Chem Rev. 2011;111: 238–250. doi:10.1021/cr100286z

19. Fujimoto K, Sawai S. A design principle of group-level decision making in cell populations. PLoS Comput Biol. 2013;9: e1003110. doi:10.1371/journal.pcbi.1003110
