# Supplementary material for: Putting theory to the test: An integrated computational/experimental chemostat model of the tragedy of the commons
Source: PLoS One. 2024 Apr 10;19(4):e0300887. doi: 10.1371/journal.pone.0300887 (PMC11006152; doi:10.1371/journal.pone.0300887)
Supplement: S2 File — (PDF) [file pone.0300887.s002.pdf]

S2. Proof that the model is well-posed

Considering the following model

$$\dot{S} = D(S^0 - S) - G(S,E)$$

$$\dot{P} = \sigma G(S,E) - \frac{1}{\gamma}(X_1 + X_2)F(P) - DP$$

$$\dot{E} = \eta Q(X_1)X_1F(P) - DE$$

$$\dot{X}_1 = X_1\left(\left(1 - Q(X_1)\right)F(P) - D\right)$$

$$\dot{X}_2 = X_2(F(P) - D)$$

where the functions ($F(P) = \frac{\mu_{max}P}{K_S+P}$, $G(S,E) = \frac{k_{cat}SE}{K_M+S}$, and $Q(X_1) = \frac{qX_1^n}{X_1^n+QS_{min}^n}$) and their parameters are as described in the main text. These functions have the following properties:

- $F(0) = G(0,E) = G(S,0) = Q(0) = 0$
- $F(P) > 0$ when $P > 0$; $G(S,E) > 0$ when $S > 0$ and $E > 0$; $Q(X_1) > 0$ when $X_1 > 0$
- $\frac{dF}{dP}(P) > 0$ when $P > 0$; $\frac{dQ}{dX_1}(X_1) > 0$ when $X_1 > 0$
- $\frac{\partial G}{\partial S}(S,E) > 0$ and $\frac{\partial G}{\partial E}(S,E) > 0$ when $S > 0$ and $E > 0$

This means there is no nutrient uptake in the absence of product and that there is no substrate-enzyme catalysis in the absence of either enzyme or substrate. That when product, enzyme, substrate, and cooperators are present, their respective functions are positive, continuous, and increasing.

It is possible to scale out the conversion factors and parameters by setting

$$x_i = \frac{X_i}{\gamma}, e = \frac{E}{\eta\gamma}, p = \frac{P}{\sigma}, \text{ and } S = s.$$

Essentially, this will transform the model so that all variables are measured in the same units, and the conversion of S to s is simply aesthetic. This yields the following scaled model:

$$\dot{s} = D(S^0 - s) - G(s,e)$$

$$\dot{p} = G(s,e) - (x_1 + x_2)F(p) - Dp$$

$$\dot{e} = Q(x_1)x_1F(p) - De$$

$$\dot{x_1} = x_1\Big(\big(1 - Q(x_1)\big)F(p) - D\Big)$$

$$\dot{x_2} = x_2(F(p) - D).$$

To prove the model is well-posed we will show that it is dissipative; that is, it is forward invariant (all solutions initiated in $\mathbb{R}^5_+$ exist and remain in $\mathbb{R}^5_+$ for all $\tau > 0$) and are ultimately uniformly bounded. This simply means that for all initial conditions where the five state variables are positive real numbers, the solutions will remain positive and will ultimately be below some threshold which does not depend on the initial condition.

By design, the system is forward invariant in $\mathbb{R}^5_+$, meaning that solutions starting in $\mathbb{R}^5_+$ stay in $\mathbb{R}^5_+$ for all τ. Mathematically, this can be determined trivially by setting each state variable to 0 within its respective equation and solving. For example, setting $s = 0$ and solving for $\dot{s}$ gives $\dot{s} = ds^0 > 0$. Doing so for all equations will reveal that each expression is greater than or equal to 0.

To show dissipativity - let m be the total mass of the system: $m = s + p + e + x_1 + x_2$. Then, $\dot{m} = \dot{s} + \dot{p} + \dot{e} + \dot{x_1} + \dot{x_2} = D(S^0 - s - p - e - x_1 - x_2) = D(S^0 - m)$, which can be verified by adding up all the equations of the model. Hence, the $\limsup_{\tau \to +\infty} m(\tau) \leq S^0$and the system is thus dissipative.

□

Taken together, biologically this means that no variable can become negative or grow infinitely - which is what would be expected in a naturally occurring biological system.
