# Supplementary material for: Putting theory to the test: An integrated computational/experimental chemostat model of the tragedy of the commons
Source: PLoS One. 2024 Apr 10;19(4):e0300887. doi: 10.1371/journal.pone.0300887 (PMC11006152; doi:10.1371/journal.pone.0300887)
Supplement: S3 File — (PDF) [file pone.0300887.s003.pdf]

S3. Proof of cheater frequency saturation

Considering the following model as described in the main text:

$$
\begin{aligned}
\dot{S} &= D(S^0 - S) - G(S, E) \\
\dot{P} &= \sigma G(S, E) - \frac{1}{\gamma}(X_1 + X_2)F(P) - DP \\
\dot{E} &= \eta Q(X_1)X_1F(P) - DE \\
\dot{X}_1 &= X_1((1 - Q(X_1))F(P) - D) \\
\dot{X}_2 &= X_2(F(P) - D)
\end{aligned}
$$

We define a new variable to represent the ratio of cheaters (X_2) in the population as

$$
R = \frac{X_2}{X_1 + X_2}, R \in [0,1].
$$

Then, using the quotient rule, we get

$$
\begin{aligned}
\dot{R} &= \frac{(X_1 + X_2)\dot{X}_2 - X_2\left(\dot{X}_1 + \dot{X}_2\right)}{(X_1 + X_2)^2} \\
&= \frac{\dot{X}_2}{(X_1 + X_2)} - \frac{X_2\left(\dot{X}_1 + \dot{X}_2\right)}{(X_1 + X_2)^2}.
\end{aligned}
$$

Next, we substitute the equation for $\dot{X}_1$ and $\dot{X}_2$ into the above expression.

$$
\begin{aligned}
\dot{R} &= \frac{X_2(F(P) - D)}{X_1 + X_2} - \frac{X_2(X_1[(1 - Q(X_1))F(P) - D] + X_2[F(P) - D])}{(X_1 + X_2)^2} \\
&= Q(X_1)F(P)R(1 - R)
\end{aligned}
$$

Thus, $\dot{R} = Q(X_1)F(P)R(1 - R)$. This is a classic logistic growth equation, with a time dependent rate of growth.

Let $\mathrm{A}(t) = Q\big(X_1(t)\big)F\big(P(t)\big)$, then $\dot{R} = \mathrm{A}(t)R(1 - R)$. This is a separable equation which can be solved as follows:

$$\frac{dR}{dt} = \mathrm{A}(t)R(1-R)$$

$$\int \frac{dR}{R(1-R)} = \int A(t)dt$$

$$\int_{R(0)}^{R(t)} \frac{1}{R} + \frac{1}{1-R} dR = \int_0^t A(s)ds$$

$$(ln|R| - ln|1-R|)|_{R(0)}^{R(t)} = \int_0^t A(s)ds$$

$$\left(ln\left|\frac{R}{1-R}\right|\right)|_{R(0)}^{R(t)} = \int_0^t A(s)ds$$

$$ln\left|\frac{R(t)\big(1-R(0)\big)}{\big(1-R(t)\big)R(0)}\right| = \int_0^t A(s)ds$$

$$\frac{R(t)}{1-R(t)} = \frac{R(0)}{1-R(0)} e^{\int_0^t A(s)ds}$$

$$R(t) = \frac{R(0)e^{\int_0^t A(s)ds}}{1-R(0)+R(0)e^{\int_0^t A(s)ds}}$$

Assuming that $\mathrm{A}(t)$ is a nonzero function (equivalently, $Q(X_1(t))F\big(P(t)\big)$ is nonzero, meaning that there must be some uptake of processed nutrient, at least for some time), there are 2 possible scenarios:

Either $\int_0^\infty \mathrm{A}(s)ds$ (which is positive due to the above assumption) is finite, or it is infinite. If it is finite, and assuming that $R(0)$ is in $(0,1)$ (meaning that initially there are some cheaters, but also some cooperators), then $R(t) \to c$ as $t \to \infty$, for some positive $c < 1$. On the other hand, if $\int_0^\infty \mathrm{A}(s)ds$ is infinite, then $R(t) \to 1$ as $t \to \infty$. In other words, the cheater frequency saturates to a constant value (to either $c < 1$ or to 1) as the population approaches a Tragedy of the Commons.

□

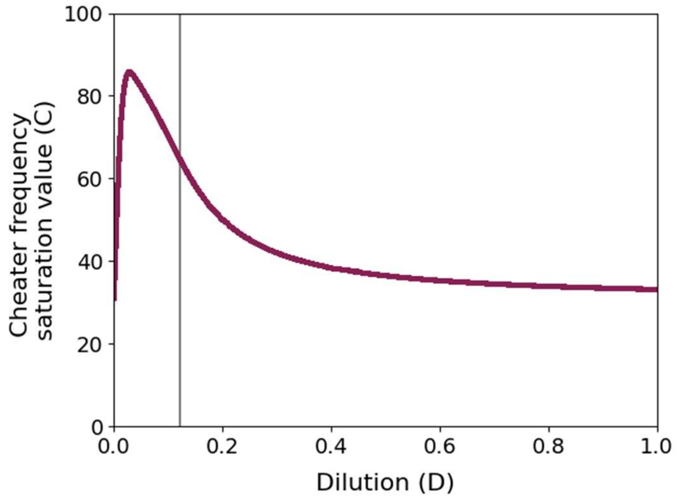

Fig S3.1. Line graph illustrating the cheater frequency saturation value (*c*) as it changes with respect to dilution rate (*D*). The vertical grey line is the dilution rate used in our experiment. *c* was determined as the simulated cheater frequency at the final experimental time point after 50 hours in batch mode and 200 hours in chemostat mode. 303 simulations were run for equidistant values of D in [0,1].
