# Supplementary material for: Putting theory to the test: An integrated computational/experimental chemostat model of the tragedy of the commons
Source: PLoS One. 2024 Apr 10;19(4):e0300887. doi: 10.1371/journal.pone.0300887 (PMC11006152; doi:10.1371/journal.pone.0300887)
Supplement: S4 File — (PDF) [file pone.0300887.s004.pdf]

S4. Sensitivity analysis of all parameters

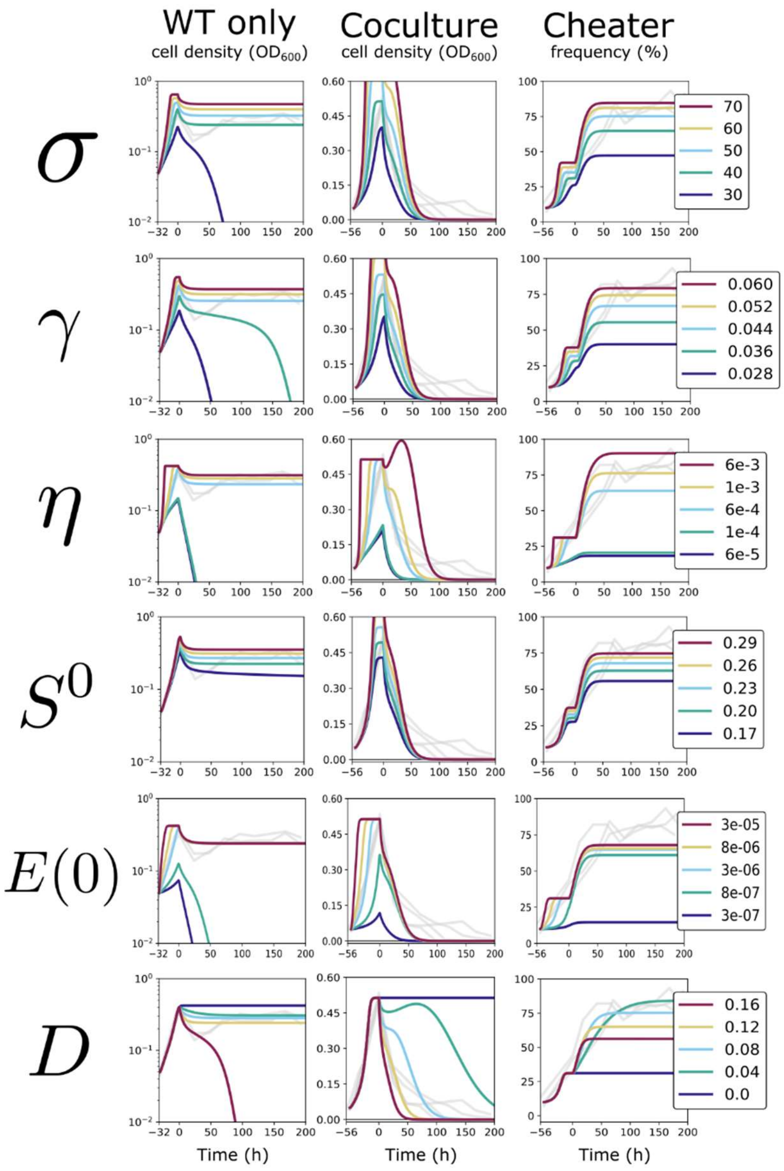

Fig S4.1: Sensitivity analysis of non-auxiliary function parameters. Five simulations were run for the parameters which are not part of an auxiliary function and the enzyme initial condition. Rows indicate the parameters or initial condition from top to bottom as follows: number of products produced per substrate molecule (σ), nutrient to biomass conversion (γ), enzyme produced per biomass (η), substrate concentration of growth medium (S^0), initial concentration of enzyme ($E(0)$), and the dilution rate (D). The columns indicate the WT-only, coculture, and cheater frequency scenarios from left to right. The experimental data are shown in grey while the simulations are shown in color with purple being the lowest simulated value and red the highest simulated value. All other parameters besides the one identified by the row heading remain as specified in Table 1 of main text. In places where all five lines are not clearly visible, they are overlapping.

Fig S4.2: Sensitivity analysis of auxiliary function parameters. Seven simulations were run for the parameters which are part of a function. Rows indicate the parameters from top to bottom as follows: metabolic burden of enzyme production (q), minimum cooperator cell density needed for quorum sensing to begin (QS_{min}), speed of transition to maximum q (n), rate of enzyme substrate turnover (k_{cat}), gelatin concentration at half-maximal growth (K_M), maximal growth rate (μ_{max}), and the concentration of product at half-maximal growth (K_S). Rows are grouped by the function they belong to from top to bottom: metabolic burden of enzyme ($Q(X_1)$), enzyme activity ($G(S,E)$), and growth rate ($F(P)$). The columns indicate the influence the parameter has on the function, followed by the WT-only, coculture, and cheater frequency scenarios from left to right. The experimental data are shown in grey while the simulations are shown in color with purple being the lowest simulated value and red the highest simulated value. All other parameters besides the one identified by the row heading remain as specified in Table 1 of main text. In places where all five lines are not clearly visible, they are overlapping.

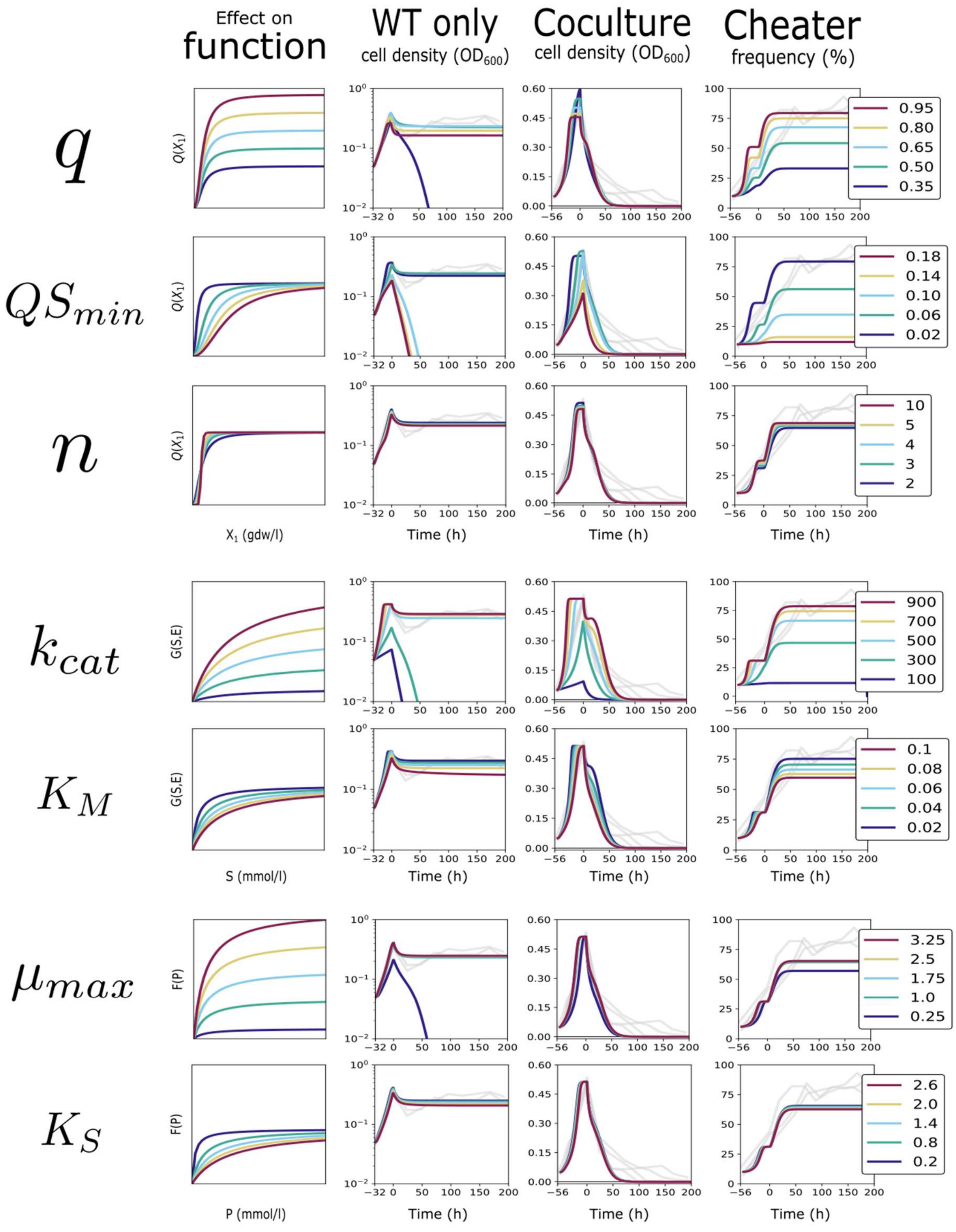

Effect on function
WT only cell density (OD_{600})
Coculture cell density (OD_{600})
Cheater frequency (%)
q
QS_{min}
n
k_{cat}
K_M
μ_{max}
K_S
$Q(X_1)$
X_1 (gdw/l)
G(S,E)
S (mmol/l)
F(P)
P (mmol/l)
Time (h)
0.95
0.80
0.65
0.50
0.35
0.18
0.14
0.10
0.06
0.02
10
5
4
3
2
900
700
500
300
100
0.1
0.08
0.06
0.04
0.02
3.25
2.5
1.75
1.0
0.25
2.6
2.0
1.4
0.8
0.2
