# Supplementary material for: Putting theory to the test: An integrated computational/experimental chemostat model of the tragedy of the commons
Source: PLoS One. 2024 Apr 10;19(4):e0300887. doi: 10.1371/journal.pone.0300887 (PMC11006152; doi:10.1371/journal.pone.0300887)
Supplement: S5 File — (PDF) [file pone.0300887.s005.pdf]

S5. Data tables

Table S5.1: Lowest RMSE and corresponding parameter values for parameter pairs†

Parameter pair	Lowest extended range RMSE (and associated parameter values)		
	WT only cell density (OD_{600})	Coculture cell density (OD_{600})	Cheater frequency (%)
σ q	0.0514 ($\sigma = 41$; $q = 0.650$)	0.0534 ($\sigma = 45$; $q = 0.375$)	8.93 ($\sigma = 63$; $q = 0.525$)
γ q	0.0513 ($\gamma = 0.043$; $q = 0.625$)	0.0536 ($\gamma = 0.048$; $q = 0.375$)	8.93 ($\gamma = 0.067$; $q = 0.525$)
QS_{min} q	0.0513 ($QS_{min}= 0.050$; $q = 0.625$)	0.0577 ($QS_{min}= 0.015$; $q = 0.275$)	8.50 ($QS_{min}= 0.0$; $q = 0.4$)
σ γ	0.0512 ($\sigma = 70$; $\gamma = 0.024$)	0.0669 ($\sigma = 30$; $\gamma = 0.072$)	9.03 ($\sigma = 66$; $\gamma = 0.034$)

† Table S5.1 contains the lowest RMSE for 3 parameter pairs across three data types: WT only, coculture, and protease deficient cheater frequency. Columns under each data type contain the lowest RMSE value and the parameter values at which the lowest RMSE occurred across their ranges. The four parameters and the ranges simulated were the number of enzymatic cleavage points (σ; [30,70]), metabolic burden (q; [0,1]), nutrient to biomass conversion (γ; [0.03,0.07]), and the minimum cooperator cell density needed for quorum sensing to begin (QS_{min}; [0,0.2]). Rows indicate the parameter pair from top to bottom as: σ and q, γ and q, and QS_{min} and q. This table corresponds to Figure 8 of the main text.

Table S5.2: *P* values from statistical analysis of emergent cheaters‡

Replicate Number	*P* value for indicated sample		
	1	2	3
Replicate 1	<0.0001	<0.0001	<0.0001
Replicate 3	0.005	>0.9999	<0.0001
Replicate 4	<0.0001	0.0002	0.0021

‡ Table S5.2 displays the *P* values from the emergent cheater coculturing data as determined by a one-way ANOVA with an *ad hoc* Bonferroni correction. Rows indicate chemostat replicates, and the sample numbers from left to right are in the same order as displayed in Figure 10 of the main text.
